# A global experimental dataset for assessing grain legume production

**DOI:** 10.1038/sdata.2016.84

**Published:** 2016-09-27

**Authors:** Charles Cernay, Elise Pelzer, David Makowski

**Affiliations:** 1 UMR Agronomie, INRA, AgroParisTech, Université Paris-Saclay, 78850 Thiverval-Grignon, France

**Keywords:** Agroecology, Environmental sciences, Biodiversity, Plant ecology

## Abstract

Grain legume crops are a significant component of the human diet and animal feed and have an important role in the environment, but the global diversity of agricultural legume species is currently underexploited. Experimental assessments of grain legume performances are required, to identify potential species with high yields. Here, we introduce a dataset including results of field experiments published in 173 articles. The selected experiments were carried out over five continents on 39 grain legume species. The dataset includes measurements of grain yield, aerial biomass, crop nitrogen content, residual soil nitrogen content and water use. When available, yields for cereals and oilseeds grown after grain legumes in the crop sequence are also included. The dataset is arranged into a relational database with nine structured tables and 198 standardized attributes. Tillage, fertilization, pest and irrigation management are systematically recorded for each of the 8,581 crop*field site*growing season*treatment combinations. The dataset is freely reusable and easy to update. We anticipate that it will provide valuable information for assessing grain legume production worldwide.

## Background & Summary

The 68th United Nations General Assembly has proclaimed 2016 as the International Year of Pulses. The Food and Agriculture Organization of the United Nations defines ‘pulses’ as plant species from the Fabaceae family cropped annually, and harvested only for dry grain (hereafter ‘grain legume’ for unambiguous use^1^). As part of this initiative, grain legumes are being promoted for use as nutritional protein-rich grains, and for their environmental and economic impacts^2–7^. Grain legumes can complement cereals as an affordable source of protein for the human diet^8–10^ and for animal feed^11–13^. Through atmospheric nitrogen fixation, grain legumes can significantly increase soil nitrogen supply and the yields of following crops^14–19^. Grain legumes can therefore play a significant role in maintaining global food security and ecosystem resilience.

Fabaceae is one of the largest families of plants worldwide, with 20,000 species growing across a wide range of climatic conditions and soil types^20,21^. Grain legume crops play significant roles in the human diet and animal feed and the environment, but only a fraction of the species in this diverse group of plants is currently exploited in agriculture. From 1961 to 2014, 75 and 90% of the area under legumes was allocated to soybean (*Glycine max*) in South America and North America, respectively^22^. Over the same period, 70, 76 and 78% of the area under legumes was covered by only three species each in Europe, Oceania and Africa: garden pea (*Pisum sativum*), soybean and beans (*Phaseolus* spp. and *Vigna* spp.) in Europe; lupins (*Lupinus* spp.), chickpea (*Cicer arietinum*) and garden pea in Oceania, and groundnut (*Arachis hypogaea*), cowpea (*Vigna unguiculata*) and beans in Africa^22^. In Asia, 76% of the area under legumes was allocated to four species (i.e., soybean, beans, groundnut and chickpea)^22^.

Experimental comparisons of grain legumes can help researchers and decision-makers to identify high-performance species with high yields. Over the last 50 years, many field experiments have assessed the agronomic and environmental performances of grain legumes. These performances vary between field sites and growing seasons, as a function of the climatic conditions and soil types. It would therefore be misleading to draw general conclusions from individual experiments considered separately. A global dataset would provide us with a unique opportunity to analyze variability in grain legume performances across a large spectrum of environmental conditions, and to rank legume species of agricultural and economic interest according to several criteria.

**Figure 1:**
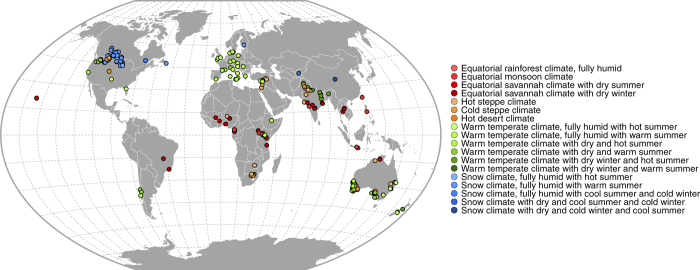
Latitude and longitude coordinates of the field sites included in the database. The Köppen-Geiger climatic classification^186^ was used to link each field site to a grid size with a resolution of 0.50 degrees of latitude by 0.50 degrees of longitude. Eighteen Köppen-Geiger climatic zones are considered: equatorial climates (red), arid climates (orange), warm temperate climates (green) and snow climates (blue). Within each main Köppen-Geiger climatic zone, each Köppen-Geiger climatic subzone is indicated by a color gradient.

We introduce here a global dataset including the results of field experiments comparing 39 grain legume species grown as sole crops. Most of grain legume species included in the database correspond to species of significant agricultural and economic importance. We have selected only experiments comparing at least two grain legume species grown at the same field site during the same growing season, to prevent any confusion between species characteristics and environmental conditions. We excluded experiments on single grain legume species because, in such experiments, differences between species can be confounded with the effects of environmental factors. Experimental data were extracted from 173 published articles^2–4,6,14–19,23–185^. In total, measurements from 360 field sites were collected across 18 Köppen-Geiger climatic zones^186^ in 41 countries (Fig. 1) over five continents (Table 1). The dataset contains 8,581 crop*field site*growing season*treatment combinations. Article references, field site locations, climatic conditions, soil types, yields, crop nitrogen contents, residual soil nitrogen contents and management practices are systematically recorded for each crop*field site*growing season*treatment combination. When available, data on non-legume species grown at the same field site during the same growing season than grain legume species, and data on non-legume species grown after grain legumes in the crop sequence are also included. Most of these non-legume species correspond to cereals and oilseeds. The data are organized into a relational database with nine structured tables and 198 standardized attributes (Tables 2 and 3 (available online only)).

The dataset can be used for two types of quantitative analysis. First, the dataset can be used to compare the crop production of a broad range of grain legume species, on the basis of experimental data with diverse criteria (e.g., grain yield, aerial biomass and crop nitrogen content). Second, the dataset can be used to assess the crop production of cereal and oilseed species following grain legume species cultivated as preceding crops in the same crop sequences, based on a consideration of field data for various criteria. The dataset is freely available to facilitate such analyses. It could easily be updated in the future, by adding the results of new experiments not originally included in the dataset. It might also be interesting to expand the dataset to include legumes grown for purposes other than grain production (e.g., forage production) or legumes grown in intercropping systems. The global dataset should prove to be a useful support for experimental assessments of the agronomic and environmental performances of a large diversity of grain legumes.

## Methods

### Literature search

We carried out a systematic search of peer-reviewed journals for articles comparing grain legume yields. We defined a grain legume species as a plant from the Fabaceae family, based on the United States Department of Agriculture Plants Database (http://plants.usda.gov/java/), and cropped for grain production. The literature search was completed on February 15, 2016. The equation search was: ‘crop* AND (legum* OR pulse*) AND (yield* OR ‘dry matter’ OR biomass) AND (compar* OR assessment OR product* OR performance*) AND (trial* OR factorial OR experiment* OR treatment* OR condition*) NOT (intercrop* OR catch OR cover OR ‘green manure’ OR forage OR fodder)’. The search terms were used to query the Institute for Scientific Information Web of Science (http://wokinfo.com/), with no restrictions concerning the date and language of publication in the article title, abstract and author keywords.

**Figure 2:**
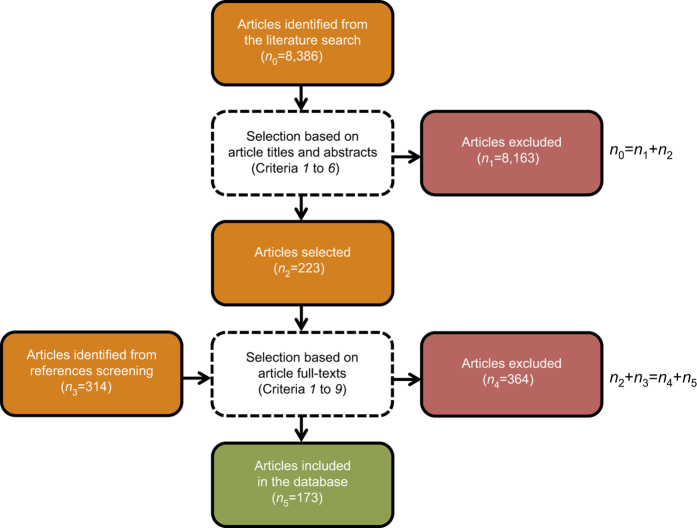
Flowchart of the steps in the literature search. Boxes with solid lines represent the articles identified (orange), excluded (red) or included in the database (green). In these boxes, the number of articles (*n*_i_) is indexed according to each step *i* of the literature search. Boxes with dashed edges represent the selection process, and selection criteria are indexed in italic.

The initial literature search identified 8,386 articles as of potential interest (Fig. 2). Each article title and article abstract were screened for eligibility according to six criteria: (1) article title and/or article abstract reporting one or several annual grain legume species grown as sole crops, (2) article title and/or article abstract reporting at least two grain legume species grown at the same field site during the same growing season, (3) article title and/or article abstract reporting at least one experiment conducted during one or several growing seasons, from the seeding stage to the harvest stage, (4) article title and article abstract referring to an article published in a peer-reviewed journal, (5) article title or article abstract written in English and (6) full-text article available. We selected 223 eligible full-text articles that met these first six criteria (Fig. 2).

Eligible full-text articles were then examined according to three additional criteria: (7) full-text article reporting raw data not duplicated in other articles or raw data that could be obtained by contacting authors, (8) full-text article reporting individual grain yield for each species and (9) full-text article reporting one or several experiments for which field site location or soil characteristics were precisely stated. We selected 60 full-text articles that met all nine criteria. This search was supplemented by screening the references cited in these 60 full-text articles. We also screened the references included in one meta-analysis about drought effects on food legume production^187^ for eligibility. When reviewing the full-text articles identified from references screening, all nine selection criteria defined above had to be met for the new article to be considered eligible. Note that, according to the criterion (2), experiments reporting data for single grain legume species were excluded. This selection criterion was used to ensure the direct comparability of different grain legume species, and avoid confounding effects between species characteristics and environmental factors. Experiments testing single species cannot be used to compare several species due to the effects of field site and growing season characteristics (e.g., climate conditions, soil types and plant diseases) on the growth and development of grain legumes.

We finally selected 173 full-text articles^2–4,6,14–19,23–185^ published between 1967 and 2016 that met all nine selection criteria (Fig. 2).

### Database structure

**Figure 3:**
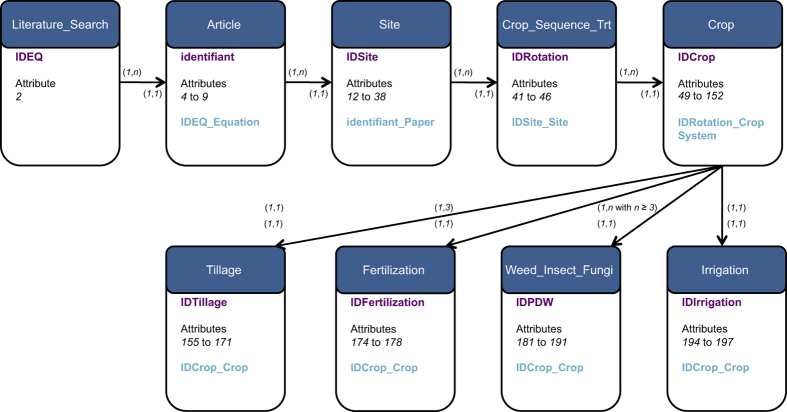
Relational model of the database. Each box represents one table. One ‘primary key’ and one ‘secondary key’ are assigned to each table (except for the ‘Literature_Search’ table, which is exclusively a ‘mother’ table). Each table includes many attributes. For the sake of readability, attributes are indexed in italic from one ‘mother’ table to one or many ‘child’ tables along the cascade path of the database (Table 3 (available online only)). Arrows indicate relationships from one ‘mother’ table to one or many ‘child’ tables. For upward and backward matching between tables, each pair of numbers in brackets indicates the cardinality of the relationships between attributes. The cardinality may involve ‘one-to-one’ (i.e., *1*,*1*) relationship or ‘one-to-many’ (i.e., *1*,*n*) relationship. For upward matching, for instance, the cardinality (*1*,*n*) from the ‘Article’ table to the ‘Site’ table indicates that one article may have one or many field sites. For backward matching, the cardinality (*1*,*1*) from the ‘Site’ table to the ‘Article’ table indicates that each field site may belong to only one article. Within each table, the names of primary and secondary keys are indicated in purple and blue, respectively.

All data are recorded in a relational database (Data Citation 1). The Structured Query Language (SQL) system is used to query and maintain the database. We used the open-access application Sequel Pro version 1.0.2 (http://www.sequelpro.com/). The data collected are grouped into nine related tables including 198 standardized attributes of five types: class, numerical, index, binary and date (Fig. 3 and Table 2). Within the database, the tables are organized according to a cascade path: each ‘child’ table is related to a ‘mother’ table. For instance, the ‘Article’ table is the ‘mother’ table for the ‘child’ ‘Site’ table (Fig. 3). The cascade path from each ‘mother’ table to each ‘child’ table is structured by a ‘primary key’ and a ‘secondary key’ (Fig. 3). A ‘primary key’ assigns an index to each row of the table, whether the table is a ‘mother’ table or a ‘child’ table. A ‘secondary key’ assigns the ‘primary key’ of a ‘mother’ table to each row of a ‘child’ table. The cardinality from each ‘mother’ table to each ‘child’ table is based on ‘one-to-one’ and ‘one-to-many’ relationships (Fig. 3).

The database is structured into nine separate but related tables, stored as CSV-formatted files (Data Citation 1). Tables are related to each other via primary and secondary keys, as explained in Fig. 3. The names, types and definitions of attributes included in the nine tables are listed in Table 3 (available online only).

The ‘Literature_Search’ table describes each step in the literature search at which each original article was selected (e.g., selection from the initial literature search or from references screening). The corresponding file is entitled ‘Literature_Search.csv’ (Data Citation 1), and includes 2 columns and 3 rows (including the row header for the names of attributes).

The ‘Article’ table describes the references of the 173 selected articles (e.g., the name of the first author and the name of the journal). The corresponding file is entitled ‘Article.csv’ file (Data Citation 1), and includes 8 columns and 174 rows (including the row header for the names of attributes).

The ‘Site’ table describes the characteristics of each field site considered in each article (e.g., latitude and longitude coordinates, soil texture, precipitation and temperature). The corresponding file is entitled ‘Site.csv’ (Data Citation 1), and includes 29 columns and 361 rows (including the row header for the names of attributes).

The ‘Crop_Sequence_Trt’ table describes each combination of crop sequences and management practices into the treatments studied at each field site (e.g., names of the species and their order in each crop sequence). The corresponding file is entitled ‘Crop_Sequence_Trt.csv’ (Data Citation 1), and includes 8 columns and 4,560 rows (including the row header for the names of attributes).

The ‘Crop’ table provides information about each crop (e.g., names of the species, seeding and harvest dates, number of replicates, grain yield, aerial biomass, crop nitrogen content, residual soil nitrogen content, water use, error terms and error types). The main attributes included in this central table are described below in the Data Records section. The corresponding file is entitled ‘Crop.csv’ (Data Citation 1), and includes 106 columns and 8,582 rows (including the row header for the names of attributes).

The ‘Tillage’ table describes tillage management for each crop (e.g., tillage tools, incorporation of preceding crop residues, seeding density and legume inoculation). The corresponding file is entitled ‘Tillage.csv’ (Data Citation 1), and includes 19 columns and 8,582 rows (including the row header for the names of attributes).

The ‘Fertilization’ table describes nitrogen, phosphate and potassium fertilizer management for each crop (e.g., names and doses of fertilizers). Only the total fertilizer dose is reported for each type of nutrient. The corresponding file is entitled ‘Fertilization.csv’ (Data Citation 1), and includes 7 columns and 25,744 rows (including the row header for the names of attributes).

The ‘Weed_Insect_Fungi’ table describes weeds, insects, and fungi management for each crop (e.g., mechanical treatment, names and doses of pesticides). The corresponding file is entitled ‘Weed_Insect_Fungi.csv’ (Data Citation 1), and includes 13 columns and 45,002 rows (including the row header for the names of attributes).

The ‘Irrigation’ table describes irrigation management for each crop (e.g., quantity of water applied and irrigation method). The corresponding file is entitled ‘Irrigation.csv’ (Data Citation 1), and includes 6 columns and 8,582 rows (including the row header for the names of attributes).

In addition to the nine CSV-formatted files (tables), downloadable from *Dryad Digital Repository* (Data Citation 1), the entire content of the database is also stored in a SQL-formatted file. The corresponding file is entitled ‘Database.sql’, and is also downloadable from *Dryad Digital Repository* (Data Citation 1). Examples of SQL queries for extracting data for each table are stored in a TXT-formatted file. The corresponding file is entitled ‘Examples_SQL_Queries.txt’, and is also downloadable from *Dryad Digital Repository* (Data Citation 1).

The names, types, and definitions of the 198 attributes included in the nine tables are reported in Table 3 (available online only).

The values (including error terms) and dates reported in graphics were digitized manually with the open-access application WebPlotDigitizer (http://arohatgi.info/WebPlotDigitizer/). The maximum error was estimated at 5.0% for the digitization of low-resolution images, generally from articles published before 1990. ‘NA’ indicates that data were ‘Not Available’ for the cell concerned. ‘NULL’ indicates a logical absence of data for attributes included in the ‘Crop’, ‘Tillage’, ‘Fertilization’, ‘Weed_Insect_Fungi’, and ‘Irrigation’ tables. For example, for the ‘Fertilization’ table, if no nitrogen fertilizer was applied to the crop (i.e., ‘0.00’ was reported in the ‘Fertilization_NPK_Dose’ attribute), then ‘NULL’ was reported for the ‘Fertilization_NPK_Dose_Product_Name’ attribute.

## Data Records

We describe below the main attributes of the ‘Crop’ table because this table includes most of the experimental data extracted from the 173 selected articles. Information on other attributes (e.g., articles, field sites, combinations of crop sequences and management practices) is defined in Table 3 (available online only).

In the ‘Crop’ table, grain yield is by far the attribute including the highest number of data. This high reporting rate reflects the explicit requirement for presence of grain yield data during the article selection process (i.e., criterion 8). Reporting rates are lower for aerial biomass, grain nitrogen content, aerial nitrogen content, fixed aerial nitrogen content, residual soil nitrogen content and water use. Table 1 presents the total number (percentage) of available and missing data for these attributes over all crop*field site*growing season*treatment combinations.

When data were not reported for some attributes (e.g., aerial biomass or water use) in the selected articles, we systematically collected data for related attributes (e.g., harvest index or grain water use efficiency) in order to retrieve the missing data. For examples, aerial biomass can be deduced from grain yield and harvest index, and water use can be deduced from grain yield and grain water use efficiency. When data were not available for any related attributes, we contacted the authors of the selected articles, and we asked them to provide us with additional raw data when available.

### ‘Crop_Sequence_Treatment_Name’ attribute

The name of each combination of crop sequences and management practices was based on the common names of the species, such as for both ‘Crop_Sequence_Trt_Name’ and ‘Crop_Sequence_Trt_Species_Order' attributes in the ‘Crop_Sequence_Trt’ table. For instance, the name of a legume-cereal sequence without application of nitrogen fertilizer (0N) could be ‘Garden pea-Common wheat, 0N’ where ‘Garden pea’ and ‘Common wheat’ are the common names listed in the United States Department of Agriculture Plants Database (http://plants.usda.gov/java/) for *Pisum sativum* and *Triticum aestivum*, respectively. Malik *et al.*^105^ and McEwen *et al.*^108^ described several crop sequences including grain legumes and crop sequences including barrelclover (*Medicago truncatula*) or common oat (*Avena sativa*), both preceding common wheat. For these two articles, we excluded the crop sequences including barrelclover and common oat because these crops were grown for forage production.

### ‘Crop_Site_Growing_Season_ID’ attribute

This attribute is an index identifying each species grown at a given field site during one or several growing seasons. Identical raw data were found to have been duplicated in two pairs of articles: Muchow *et al.*^114^ and Sinclair *et al.*^153^ on the one hand, and Heenan *et al.*^71^ and Armstrong *et al.*^2^ on the other. The duplicated raw data from Sinclair *et al.*^153^ and Heenan *et al.*^71^ were excluded because the number of crop*field site*growing season*treatment combinations was smaller in these two articles than in their duplicates.

### ‘Crop_Species_Scientific_Name’ and ‘Crop_Species_Common_Name’ attributes

These attributes give the scientific and common names of the species. The scientific name of each species was related to the common name listed in the United States Department of Agriculture Plants Database (http://plants.usda.gov/java/), to avoid confusion due to the use of different common names for the same species. In the absence of a common name for *Brassica campestris*, *Lupinus atlanticus* and *Triticum sativum*, the scientific names of these species were used as common names. In the presence of fallow period, it was not possible to give a scientific name and a common name, and ‘Fallow’ was reported.

### ‘Crop_Date_From_Seeding_To_Harvest_Day_Number’ attribute

We calculated the number of days from seeding date to harvest date, with the open-access application Time and Date (http://www.timeanddate.com/). For data averaged across multiple growing seasons, we calculated the number of days from seeding date to harvest date for each growing season and then obtained the average by dividing by the total number of growing seasons.

Some articles approximated seeding date and harvest date by describing these events as occurring in the ‘early’, ‘middle’ or ‘late’ part of the month. We defined ‘early’ as the first 15-day period of the month (1st–15th), ‘middle’ as the 15th day of the month and ‘last’ as the second 15-day period of the month (15th–30th or 15th–31st). In these cases, the number of days from seeding to harvest was calculated by selecting the last day of the period concerned, i.e., the 15th day of the month for ‘early’ and ‘middle’ and the 30th or 31st day of the month for ‘late’.

Some articles reported only the number of days from seeding to harvest, without indicating precise dates or months. In these cases, we reported only the number of days from seeding to harvest. We used the expression ‘NA NA NA’ (i.e., ‘Day Month Year’ formatted expression) for both seeding and harvest dates.

### ‘Crop_Following_Number’ attribute

This attribute is used to distinguish preceding crops from following crops in the crop sequence. It takes three values: ‘0’ (i.e., the main crop or the preceding crop, mostly grain legumes), ‘1’ (i.e., the following crop, mostly cereals and oilseeds) and ‘2’ (i.e., the crop after the following crop, mostly cereals and oilseeds).

### ‘Crop_Multiple_Following_For_Same_Preceding’ attribute

Some studies reported results for many different crops and management practices following the same preceding crop. The binary ‘Crop_Multiple_Following_For_Same_Preceding’ attribute was used to identify data associated with the same preceding crop.

### ‘Crop_Across_Treatment_Averaged_Value’ and ‘Crop_Across_Treatment_Averaged_Value_Type’ attributes

For species grown at the same field site during the same growing season, some articles reported only data averaged over combinations of treatments (e.g., cultivar*seeding date*presence of irrigation). We included these data provided that each type of individual treatment was precisely defined in the article. In all cases, we systematically reported whether or not the data were averaged over combinations of treatments. When data were averaged over combinations of treatments, the total number of replicates was calculated as the sum of the replicates for each of the treatments for which results were averaged.

For articles reporting data for several cultivars of the same species but without data averaging, the data were reported separately for each cultivar. For articles reporting data averaged over several cultivars of the same species, only the averaged data were included in the dataset. The total number of replicates was calculated by multiplying the number of replicates of each cultivar by the total number of cultivars.

### ‘Crop_Across_Species_Same_Treatment_Value’ and ‘Crop_Across_Species_Same_Treatment_Value_Type’ attributes

In some articles, different types of treatment were applied to species grown at the same site during the same growing season. Each different type of treatment was reported in this case.

### ‘Crop_Replicate_Number’ attribute

As mentioned above, when averaged data were reported in the articles, the number of replicates was equal to the sum of the replicates used to calculate each average.

### ‘Crop_Yield_Grain’ attribute

**Figure 4:**
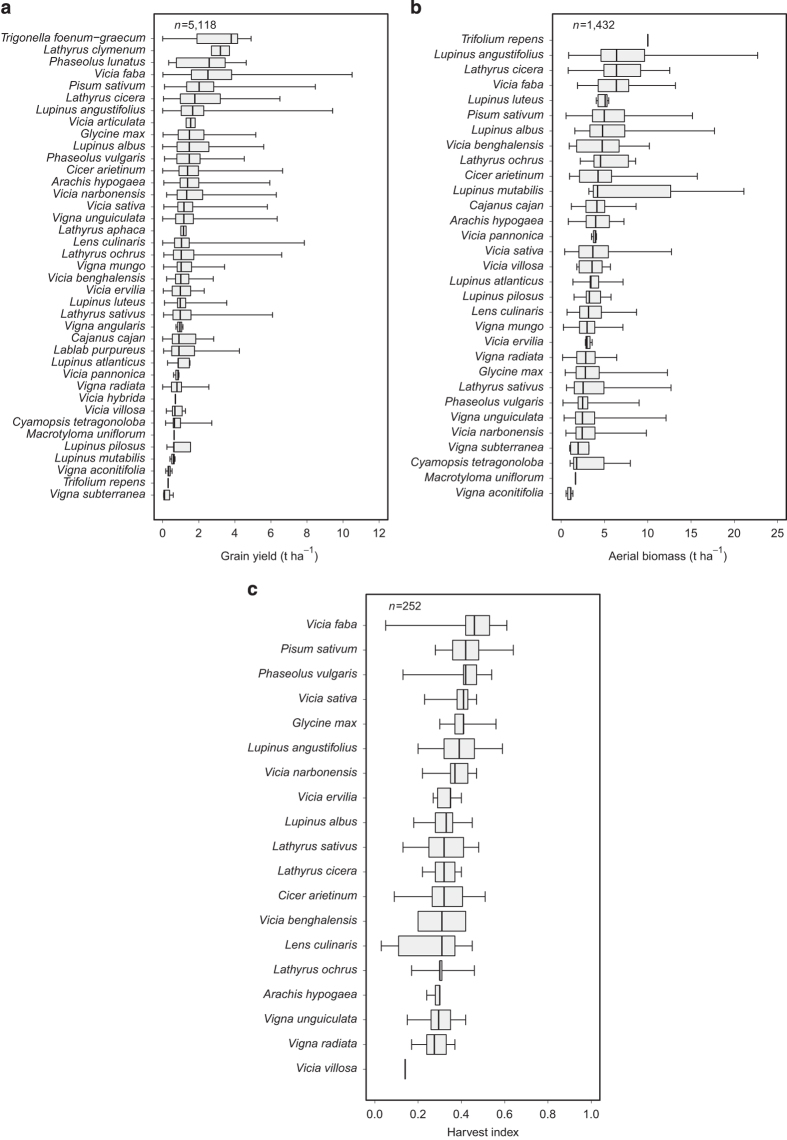
Distribution of grain yield (t ha^−1^) for 39 grain legume species (**a**), aerial biomass (t ha^−1^) for 31 grain legume species (**b**), and harvest index for 19 grain legume species (**c**). Distributions are derived using data extracted from the database without additional calculations. Intrabox lines indicate medians, box edges indicate 25th and 75th percentiles, and whiskers indicate minimum and maximum values. The number of observations (*n*) is also indicated. The scientific names of the species are ranked in descending order of median values.

This attribute corresponds to grain yield data, with a few exceptions. For *Brassica chinensis* (pak choi), *Citrullus lanatus* (watermelon), *Gossypium hirsutum* (upland cotton), *Ipomoea batatas* (sweet potato) and *Solanum lycopersicum* (garden tomato), the yields reported are the economic yields. For *Arachis hypogaea* (peanut), pods are included in grain yields. In all other situations, the yield data given correspond to grain yields. Mutant non-nodulating legume cultivars, shading treatment and under-sowing treatment were excluded from the database. When grain yield data of following crops were confounded between the effect of preceding species and the effect of nitrogen fertilizer dose, these data were also excluded. Data were reported in 96% of all crop*field site*growing season*treatment combinations. Grain yield varied strongly both between grain legume species and between articles for a given species (Fig. 4a). Median grain yield was lowest for *Vigna subterranea* (bambarra groundnut) and highest for *Trigonella foenum*-*graecum* (sicklefruit fenugreek).

### ‘Crop_Biomass_Aerial’ attribute

This attribute corresponds to aerial biomass data. Data were reported in 27% of all crop*field site*growing season*treatment combinations. Aerial biomass varied considerably both between grain legume species and between articles for a given species (Fig. 4b). Median aerial biomass was lowest for *Vigna aconitifolia* (moth bean) and highest for *Trifolium repens* (white clover).

### ‘Crop_Yield_Grain_DM_Percentage’ and ‘Crop_Biomass_Aerial_DM_Percentage’ attributes

These two attributes correspond to the percentage of dry matter to which grain yield and aerial biomass correspond, respectively. When only the percentage of dry matter corresponding to aerial biomass was available and grains were included in aerial biomass, we assumed that the grains accounted for the same percentage of dry matter as the aerial biomass.

### ‘Crop_Harvest_Index’ attribute

This attribute was reported in the database to calculate aerial biomass at physiological maturity from grain yield. Data were reported in 4% of all crop*field site*growing season*treatment combinations (Fig. 4c). Median harvest index was lowest for *Vicia villosa* (winter vetch) and highest for *Vicia faba* (fababean).

### ‘Crop_N_Quantity_Grain’ and ‘Crop_N_Quantity_Aerial’ attributes

**Figure 5:**
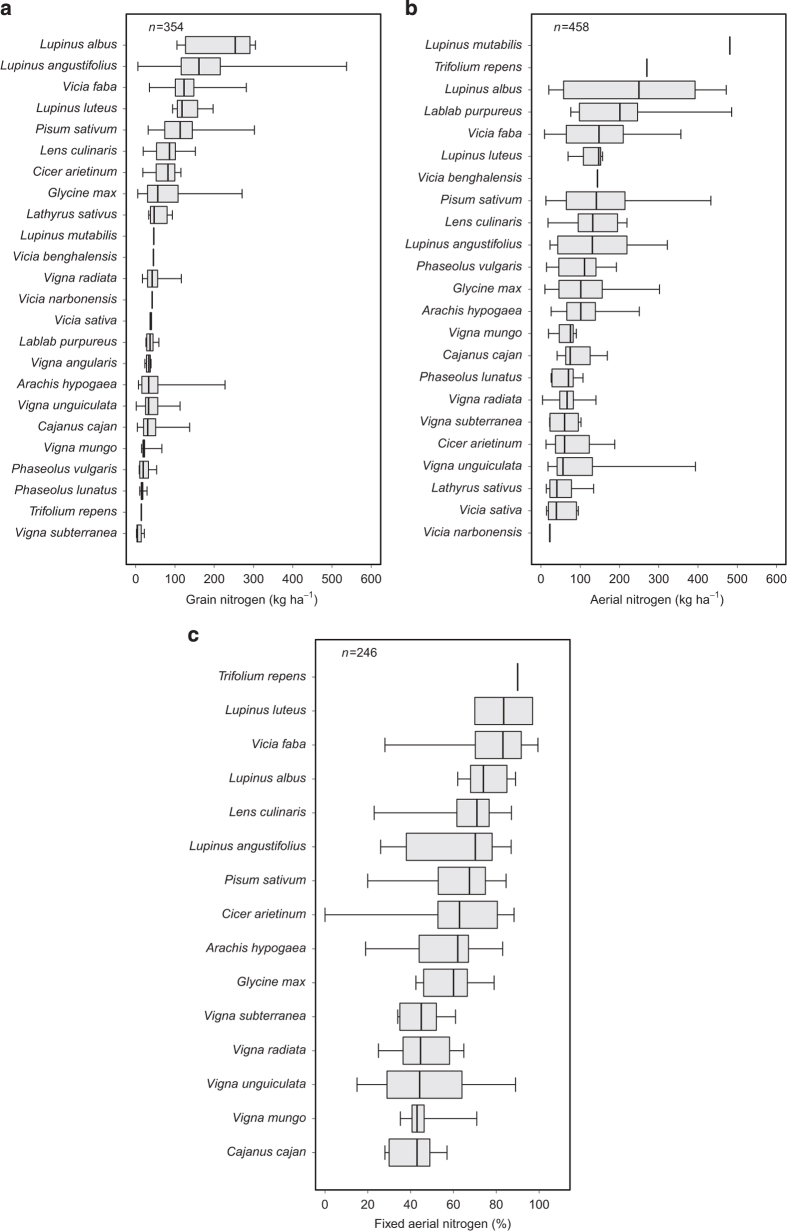
Distribution of grain nitrogen (kg ha^−1^) for 24 grain legume species (**a**), aerial nitrogen (kg ha^−1^) for 23 grain legume species (**b**), and fixed aerial nitrogen (%) for 15 grain legume species (**c**). Distributions are derived using data extracted from the database without additional calculations. Intrabox lines indicate medians, box edges indicate 25th and 75th percentiles, and whiskers indicate minimum and maximum values. The number of observations (*n*) is also indicated. The scientific names of the species are ranked in descending order of median values.

These two attributes correspond to the quantity of nitrogen in grains and aerial components, respectively. For the ‘Crop_N_Quantity_Grain’ attribute, data were reported in 10% of all crop*field site*growing season*treatment combinations. For the ‘Crop_N_Quantity_Aerial’ attribute, data were reported in 10% of all crop*field site*growing season*treatment combinations. As previous attributes, grain and aerial nitrogen quantities varied both between grain legume species and between articles for a given species (Fig. 5a,b). Median grain nitrogen quantity was lowest for *Vigna subterranea* (bambarra groundnut) and highest for *Lupinus albus* (white lupine). Median aerial nitrogen quantity was lowest for *Vicia narbonensis* (purple broad vetch) and highest for *Lupinus mutabilis* (sweet tarwi).

### ‘Crop_N_Fixed_Percentage_Aerial’ attribute

This attribute corresponds to the percentage of aerial nitrogen fixed by legume species. ‘NA’ was systematically reported for non-legume species. Data were reported in 3% of all crop*field site*growing season*treatment combinations (Fig. 5c). Median fixed aerial nitrogen percentage was lowest for *Cajanus cajan* (pigeonpea) and highest for *Trifolium repens* (white clover).

### ‘Crop_N_Fixed_Percentage_Aerial_Method’ and ‘Crop_N_Fixed_Percentage_Aerial_Reference_Species’ attributes

These two attributes correspond to the method used to determine the percentage of aerial nitrogen fixed by legume species (e.g., the ^15^N isotope dilution method or the A-value method), and the scientific name of the non-fixing reference species. Some articles used a legume reference species rather than a non-legume reference species. In all cases, the legume reference species was a mutant non-nodulating legume cultivar that did not fix atmospheric nitrogen.

### ‘Crop_Biomass_Aerial_Stage_Detailed’, ‘Crop_Biomass_Aerial_Stage_Simplified’, ‘Crop_N_Fixed_Percentage_Aerial_Stage_Detailed’ and ‘Crop_N_Fixed_Percentage_Aerial_Stage_Simplified’ attributes

These attributes correspond to the phenological stages at which aerial biomass and the percentage of fixed aerial nitrogen (or the quantity of fixed aerial nitrogen with the ‘Crop_N_Fixed_Quantity_Aerial’ attribute) were determined. The ‘Crop_Biomass_Aerial_Stage_Detailed’ and ‘Crop_N_Fixed_Percentage_Aerial_Stage_Detailed’ attributes correspond to the detailed phenological stage originally stated in the article. The ‘Crop_Biomass_Aerial_Stage_Simplified’ and ‘Crop_N_Fixed_Percentage_Aerial_Stage_Simplified’ attributes correspond to a simplified phenological stage divided into ‘Before physiological maturity’ and ‘Physiological maturity’.

### ‘Crop_Protein_Quantity_Percentage_Grain’ attribute

This attribute corresponds to the percentage or the quantity of protein in grains. In the selected articles, these protein contents were often calculated by multiplying the percentage or the quantity of nitrogen in grains by a constant. However, this constant differed between articles. Note that only a few articles referred to the percentage or the quantity of protein. We reported the percentage or the quantity of protein in grains independently of the percentage or the quantity of nitrogen in grains.

### ‘Crop_N_Balance_Simplified’ attribute

This attribute corresponds to the simplified nitrogen balance originally calculated in the articles (e.g., the difference between the quantity of nitrogen in grains and the quantity of fixed aerial nitrogen). Nitrogen balance data were only reported if the attributes used to calculate them were not directly available from raw data (e.g., the quantity of nitrogen in grains and the quantity of fixed aerial nitrogen). This was the case for only three articles.

### ‘Crop_N_Soil_Quantity_Percentage_Seeding’ and ‘Crop_N_Soil_Quantity_Percentage_Harvest’ attributes

These two attributes correspond to the percentage or the quantity of soil nitrogen at seeding and at harvest, respectively.

### ‘Crop_N_Soil_Quantity_Percentage_Seeding_Type’, ‘Crop_N_Soil_Quantity_Percentage_Seeding_Depth’, ‘Crop_N_Soil_Quantity_Percentage_Seeding_Date’, ‘Crop_N_Soil_Quantity_Percentage_Harvest_Type’, ‘Crop_N_Soil_Quantity_Percentage_Harvest_Depth’ and ‘Crop_N_Soil_Quantity_Percentage_Harvest_Date’ attributes

These attributes correspond to (i) the type of nitrogen (e.g., nitrogen or nitrate or mineral), (ii) the depth of soil used to determine the percentage or the quantity of soil nitrogen and (iii) the date at which soil measurements were made. These attributes were reported at both seeding and harvest.

### ‘Crop_Water_Use_Balance’ attribute

**Figure 6:**
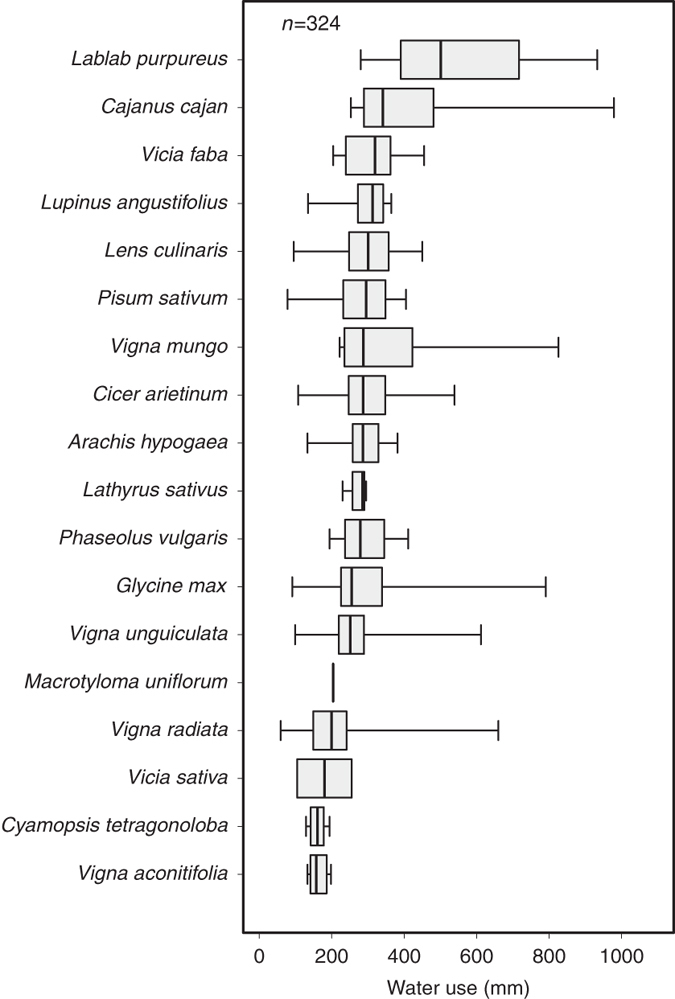
Distribution of water use (mm) for 18 grain legume species. Water use is calculated using different types of equations, indicated within the ‘Crop_Water_Use_Balance_Equation’ attribute. The distribution is derived using data extracted from the database without additional calculations. Intrabox lines indicate medians, box edges indicate 25th and 75th percentiles, and whiskers indicate minimum and maximum values. The number of observations (*n*) is also indicated. The scientific names of the species are ranked in descending order of median values.

This attribute corresponds to the water use or the water balance, according to the equation given in the selected articles. Data were reported in 6% of all crop*field site*growing season*treatment combinations. Water use (or water balance) varied both between grain legume species and between articles for a given species (Fig. 6). Median water use (or water balance) was lowest for *Vigna aconitifolia* (moth bean) and highest for *Lablab purpureus* (hyacinthbean).

### ‘Crop_Harvest_Index’, ‘Crop_N_Percentage_Grain’, ‘Crop_N_Percentage_Aerial’, ‘Crop_N_Harvest_Index’, ‘Crop_N_Fixed_Quantity_Aerial’, ‘Crop_Water_Use_Balance_Efficiency_Grain’ and ‘Crop_Water_Use_Balance_Efficiency_Aerial’ attributes

These seven attributes were reported in the database to calculate missing data: aerial biomass, quantity of nitrogen in grains, quantity of nitrogen in aerial components, percentage of fixed aerial nitrogen, and water use.

### ‘Crop_Biomass_Aerial_Definition’, ‘Crop_N_Percentage_Aerial_Definition’, ‘Crop_N_Quantity_Aerial_Definition’, ‘Crop_N_Fixed_Quantity_Aerial_Definition’ and ‘Crop_Water_Use_Balance_Efficiency_Aerial_Definition’ attributes

Different aerial components were included in the aerial biomass, the percentage or the quantity of aerial nitrogen, and the efficiency of aerial water use or aerial water balance. These five attributes were used to determine the aerial components originally reported in the articles. When the ‘shoot’, ‘straw’ and ‘stubble’ terms were used to define the aerial components in the articles, we assumed that the grains were not included in the aerial components. This information was reported for (i) the aerial biomass in the ‘Crop_Biomass_Aerial_Definition’ attribute, (ii) the percentage of aerial nitrogen in the ‘Crop_N_Percentage_Aerial_Definition’ attribute, (iii) the quantity of aerial nitrogen in the ‘Crop_N_Quantity_Aerial_Definition’ attribute, (iv) the quantity of fixed aerial nitrogen in the ‘Crop_N_Fixed_Quantity_Aerial_Definition’ attribute, and (v) the efficiency of aerial water use or aerial water balance in the ‘Crop_Water_Use_Balance_Efficiency_Aerial_Definition’ attribute.

### ‘Crop_N_Balance_Simplified_Equation’ and ‘Crop_Water_Use_Balance_Equation’ attributes

For these two attributes, we reported the equations used to calculate simplified nitrogen balance and water use or water balance, respectively.

### Attributes relating to error terms and error types

When available, we systematically reported error terms and error types associated with data about grain yield, aerial biomass, crop nitrogen content, residual soil nitrogen content and water use. For the ‘Crop_Yield_Grain’ attribute, the ‘Crop_Yield_Grain_Error’ attribute indicates the error term and the ‘Crop_Yield_Grain_Error_Type’ attribute indicates the error type for a given item of grain yield data for a given crop in the ‘Crop’ table. Error terms and error types were reported as raw data. For instance, when an article reported the error type as Fisher's Least Significant Difference, the data were directly reported as Fisher's Least Significant Difference. Unidentified error bars digitized from graphs were assumed to represent standard errors. When available, the numbers of replicates were also reported. For 48% of grain yields, both error terms and the numbers of replicates were reported. For 47% of grain yields, only the number of replicates was reported.

## Technical Validation

Each article was read carefully at least three times by the same person, to determine the type and the quantity of data reported by the authors. Once the data had been extracted, all the data reported in the tables were checked at least three times by the same person, to identify possible mistakes. SQL subset queries were systematically performed, to check the structural validity and coherence of class, numerical, index, binary and date attributes within each table, and to check the relationships between ‘mother’ and ‘child’ tables. Once the set of data was complete, SQL queries were carried out, to compare the entire content of the database with the original data reported in the selected articles. We systematically and manually checked for outliers in order to detect possible mistakes made during data extraction. We returned to the original articles as many times as needed to check the accuracy of the data. We checked the qualitative and quantitative contents of all class, numerical, index, binary and date attributes by importing each table in turn into the R software (version 3.2, https://cran.r-project.org/), and by visualizing data distribution for each attribute in turn. When the meaning of the data reported in the articles was unclear, authors were directly contacted and asked to provide additional information about their experimental protocols. Authors were also asked to provide additional data, particularly if large numbers of treatments had been averaged in their articles. Overall, 17 authors provided us with additional information and raw data (see the Acknowledgements section).

## Usage Notes

The dataset is based on a compilation of experimental data published in 173 articles over the last 50 years. To our knowledge, this dataset is unique and constitutes the most comprehensive agronomic dataset for grain legume crops worldwide.

The dataset can be analyzed to assess performances for a broad diversity of grain legume species, and to provide global rankings for these species in terms of grain yield, aerial biomass, harvest index, aerial nitrogen fixation, nitrogen content in aerial components, nitrogen balance, and water use. It can also be used to assess the effect of including different grain legumes as preceding crops, before cereals and oilseeds in the same crop sequences. Global species rankings were recently estimated for energy crops^188^, but never for grain legumes. Rankings of grain legume species could be directly derived from our dataset by using standard meta-analysis methods based on random-effect models^188^. Attributes describing environmental factors (e.g., climate conditions and soil types) and management practices (e.g., tillage, fertilization, pest management and irrigation) can be used to analyze the variability of grain legume performances over field sites, growing seasons, and management practices.

Our dataset covers several contrasted geographical areas. It can be used to target suitable grain legume species for cultivation in particular pedoclimatic conditions. In the context of climate change, the database represents a useful resource to assess comparatively the production of grain legume species in drought-prone environments, or to identify innovative agricultural techniques for improving grain legume cultivation under yield-limiting abiotic and biotic stresses.

**Figure 7:**
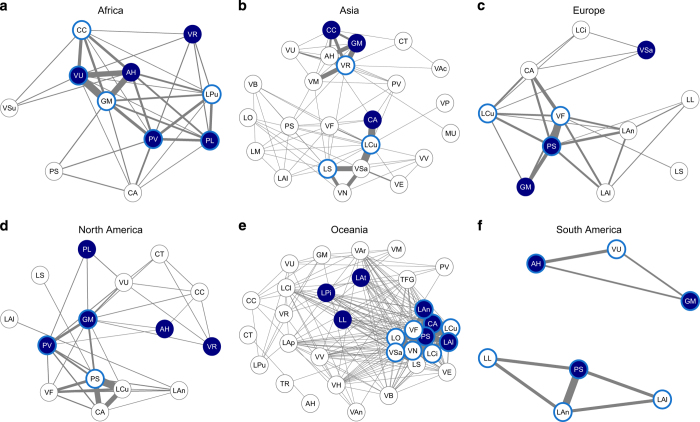
Regional networks of grain legume species included in the database. The regions considered are: (**a**) Africa, (**b**) Asia, (**c**) Europe, (**d**), North America (**e**) Oceania, and (**f**) South America. The links represent the pairs of species grown simultaneously at the same field sites during the same growing seasons. The thickness of the links increases with the number of field sites and the number of growing seasons over which the species are compared. The three most widely cropped grain legume species in each region over the 1961–2014 period, according to the crop classification and crop data from the Statistics Division of Food and Agriculture Organization of the United Nations^22^, are indicated as nodes in dark blue. The three most frequently compared grain legume species in the experimental dataset are indicated, by region, with light blue edges. The scientific names of grain legume species are abbreviated: AH, *Arachis hypogaea*; CA, *Cicer arietinum*; CC, *Cajanus cajan*; CT, *Cyamopsis tetragonoloba*; GM, *Glycine max*; LAl, *Lupinus albus*; LAn, *Lupinus angustifolius*; LAp, *Lathyrus aphaca*; LAt, *Lupinus atlanticus*; LCi, *Lathyrus cicera*; LCl, *Lathyrus clymenum*; LCu, *Lens culinaris*; LL, *Lupinus luteus*; LM, *Lupinus mutabilis*; LO, *Lathyrus ochrus*; LPi, *Lupinus pilosus*; LPu, *Lablab purpureus*; LS, *Lathyrus sativus*; MU, *Macrotyloma uniflorum*; PL, *Phaseolus lunatus*; PS, *Pisum sativum*; PV, *Phaseolus vulgaris*; TFG, *Trigonella foenum-graecum*; TR, *Trifolium repens*; VAc, *Vigna aconitifolia*; VAn, *Vigna angularis*; VAr, *Vicia articulata*; VB, *Vicia benghalensis*; VE, *Vicia ervilia*; VF, *Vicia faba*; VH, *Vicia hybrida*; VM, *Vigna mungo*; VN, *Vicia narbonensis*; VP, *Vicia pannonica*; VR, *Vigna radiata*; VSa, *Vicia sativa*; VSu, *Vigna subterranea*; VU, *Vigna unguiculata*; VV, *Vicia villosa*.

Subsets of the dataset can be used to address regional issues. Figure 7 presents six regional networks including the pairs of grain legume species frequently compared at the same field sites during the same growing seasons, and the grain legume species that were not frequently compared with each other. Such networks can be used to identify the species for which reliable comparisons are feasible, and those for which limited data are available. A quantitative analysis can then be computed to determine regional rankings of grain legume species. This approach could be used to identify highly productive species, and to compare them with major regional grain legume crops (e.g., garden pea in Europe or soybean in North America). Our dataset could thus shed new light on the potential value of as yet underused grain legumes from regional to global scales.

As geographical coordinates of the experiments were systematically reported, our dataset can be connected to large-scale climate and soil maps, and to Geographic Information Systems. An example is shown in Fig. 1 where the Köppen-Geiger climatic classification was indicated for field sites included in the database. Similar maps could be easily produced using other global classification of agroecological zones (e.g., the Global Agro-Ecological Zones Data Portal, http://gaez.fao.org/Main.html#), or soil typology (e.g., the Soils Portal of the Food and Agriculture Organization of the United Nations, http://www.fao.org/soils-portal/soil-survey/soil-maps-and-databases/harmonized-world-soil-database-v12/en/).

The dataset is also useful for comparing productivity levels of native and non-native grain legume species used as raw materials for food and feed across diverse geographic regions. Grain yield data can be converted into crude protein or energy contents metabolizable for livestock animals (e.g., pigs and poultry) using, for example, the Feedipedia Animal Feed Resources Information System (http://www.feedipedia.org/).

In the future, the dataset could be expanded in different ways. Results of new experiments comparing grain legume species can be easily included in our database. So far, we focused on legume species produced for grains, but legume grown for forage can also be included in the database without changing the relational database structure. In many world regions such as Africa, Asia and South America, agricultural grain legumes are frequently intercropped. Data collected in intercropping experiments could be further included in our dataset. Note that the relational structure of the database is relatively coercive, and should be modified with great care. The addition of a new table can have consequences on the relational framework and the cardinality relationships. But new data or new attributes can be easily incremented in existing tables.

The CSV format is well adapted for analyzing data using standard statistical softwares such as the R software (https://cran.r-project.org/). However, because of the cascade path between tables and of the cardinality relationships between attributes (Fig. 3), data extraction can be easily performed using SQL queries. An example of query is presented below for extracting binary data indicating absence (‘0’) or presence (‘1’) of tillage management for grain legume species included in the article indexed ‘29’ in our dataset:

SELECT IDCrop, Crop_Species_Scientific_Name, IDTillage, Tillage_Presence_Tillage

FROM Article, Site, Crop_Sequence_Trt, Crop, Tillage

WHERE identifiant=identifiant_Paper

AND IDSite=IDSite_Site

AND IDRotation=IDRotation_CropSystem

AND IDCrop=Tillage.IDCrop_Crop

AND identifiant='29'

The result of the SQL query is:

IDCrop, Crop_Species_Scientific_Name, IDTillage, Tillage_Presence_Tillage


853     Cicer arietinum     849     1
854     Vicia faba          851     1
857     Lens culinaris      856     1
858     Pisum sativum       858     1
859     Cicer arietinum     860     1
860     Vicia faba          861     1
861     Lens culinaris      862     1
862     Pisum sativum       863     1
864     Cicer arietinum     864     1
865     Vicia faba          865     1
866     Lens culinaris      866     1
867     Pisum sativum       867     1
869     Cicer arietinum     868     1
870     Vicia faba          870     1
871     Lens culinaris      871     1
872     Pisum sativum       872     1
873     Cicer arietinum     873     0
874     Vicia faba          874     0
875     Lens culinaris      875     0
876     Pisum sativum       876     0
877     Cicer arietinum     877     0
878     Vicia faba          878     0
879     Lens culinaris      879     0
880     Pisum sativum       880     0
881     Cicer arietinum     881     0
882     Vicia faba          882     0
883     Lens culinaris      883     0
884     Pisum sativum       884     0
885     Cicer arietinum     885     0
887     Vicia faba          886     0
888     Lens culinaris      887     0
890     Pisum sativum       889     0  


Other examples of SQL queries are shown in the TXT-formatted file entitled ‘Examples_SQL_Queries.txt’, downloadable from *Dryad Digital Repository* (Data Citation 1).

## Additional Information

**How to cite this article:** Cernay, C. *et al.* A global experimental dataset for assessing grain legume production. *Sci. Data* 3:160084 doi: 10.1038/sdata.2016.84 (2016).

## Supplementary Material



## Figures and Tables

**Table 1:** Number (percentage) of field sites, field site*growing season and field site*growing season*treatment combinations, and data for grain yield, aerial biomass, grain nitrogen content, aerial nitrogen content, fixed aerial nitrogen content, residual soil nitrogen content and water use, by main world regions.

**Region**	**Field site**	Field site*growing season	Field site*growing season*treatment	**Grain yield**	**Aerial biomass**	**Grain nitrogen content**	**Aerial nitrogen content**	**Aerial fixed nitrogen content**	**Residual soil nitrogen content**	**Water use**
Oceania	131 (36.39)	183 (22.45)	2,372 (27.64)	2,324 (28.25)	727 (27.16)	191 (19.47)	107 (12.53)	28 (7.11)	216 (11.71)	142 (16.01)
North America	72 (20.00)	165 (20.25)	2,597 (30.26)	2,524 (30.68)	600 (22.41)	285 (29.05)	178 (20.84)	38 (9.64)	806 (43.69)	474 (53.44)
Asia	65 (18.06)	253 (31.04)	1,475 (17.19)	1,408 (17.11)	598 (22.34)	129 (13.15)	171 (20.02)	87 (22.08)	468 (25.37)	259 (29.20)
Africa	48 (13.33)	101 (12.39)	907 (10.57)	827 (10.05)	243 (9.08)	161 (16.41)	172 (20.14)	145 (36.80)	70 (3.79)	0 (0.00)
Europe	39 (10.83)	102 (12.52)	1,174 (13.68)	1,089 (13.24)	479 (17.89)	181 (18.45)	188 (22.01)	74 (18.78)	255 (13.82)	12 (1.35)
South America	5 (1.39)	11 (1.35)	56 (0.65)	56 (0.68)	30 (1.12)	34 (3.47)	38 (4.45)	22 (5.58)	30 (1.63)	0 (0.00)
Total number (percentage) of available data	360 (100.00)	815 (100.00)	8,581 (100.00)	8,228 (95.89)	2,677 (31.20)	981 (11.43)	854 (9.95)	394 (4.59)	1,845 (21.50)	887 (10.33)
Total number (percentage) of missing data	0 (0.00)	0 (0.00)	0 (0.00)	353 (4.11)	5,904 (68.80)	7,600 (88.57)	7,727 (90.05)	8,187 (95.41)	6,736 (78.50)	7,694 (89.66)
Total number (percentage) of data	360 (100.00)	815 (100.00)	8,581 (100.00)	8,581 (100.00)	8,581 (100.00)	8,581 (100.00)	8,581 (100.00)	8,581 (100.00)	8,581 (100.00)	8,581 (100.00)
Regions are ranked in descending order of field sites. Grain yield includes data from the ‘Crop_Yield_Grain’ attribute. Aerial biomass includes data from both ‘Crop_Biomass_Aerial’ and ‘Crop_Harvest_Index’ attributes. Grain nitrogen content includes data from both ‘Crop_N_Quantity_Grain’ and ‘Crop_N_Percentage_Grain’ attributes. Aerial nitrogen content includes data from the ‘Crop_N_Quantity_Aerial’, ‘Crop_N_Percentage_Aerial’ and ‘Crop_N_Harvest_Index’ attributes. Fixed aerial nitrogen content includes data from both ‘Crop_N_Fixed_Quantity_Aerial’ and ‘Crop_N_Fixed_Percentage_Aerial’ attributes. Residual soil nitrogen content includes data from both ‘Crop_N_Soil_Quantity_Percentage_Seeding’ and ‘Crop_N_Soil_Quantity_Percentage_Harvest’ attributes. Water use includes data from the ‘Crop_Water_Use_Balance’, ‘Crop_Water_Use_Balance_Efficiency_Grain’ and ‘Crop_Water_Use_Balance_Efficiency_Aerial’ attributes. The total number (percentage) of available data and the total number (percentage) of missing data are calculated over all considered world regions.										

**Table 2:** Number (percentage) of attribute types included in the nine tables of the database.

**Table**	**Class attribute**	**Numerical attribute**	**Index attribute**	**Binary attribute**	**Date attribute**	**Total**
Literature_Search	1	0	1	0	0	2 (1.01)
Article	5	0	2	0	1	8 (4.04)
Site	11	12	2	0	4	29 (14.65)
Crop_Sequence_Trt	3	2	2	1	0	8 (4.04)
Crop	47	41	3	5	10	106 (53.54)
Tillage	7	6	2	4	0	19 (9.60)
Fertilization	4	1	2	0	0	7 (3.54)
Weed_Insect_Fungi	2	1	2	6	2	13 (6.57)
Irrigation	2	1	2	1	0	6 (3.03)
Total	82 (41.41)	64 (32.32)	18 (9.09)	17 (8.59)	17 (8.59)	198 (100.00)
Tables are presented according to the cascade path of the database.						

**Table 3:** Tables and attributes included in the dataset

**Table number**	**Table name**	**Attribute number**	**Attribute name**	**Attribute type**	**Attribute definition**
1	Literature_Search	1	IDEQ	Index	Index of the step of literature search. Primary key of the ‘Literature_Search’ table.
1	Literature_Search	2	Literature_Search_Origin	Class	Step of the literature search.
2	Article	3	identifiant	Index	Index of each article from each step of the literature search. Primary key of the ‘Article’ table.
2	Article	4	Article_Author_First	Class	Name of the first author.
2	Article	5	Article_Title	Class	Article title.
2	Article	6	Article_Year_Publication	Date	Publication year or ‘NA’.
2	Article	7	Article_Journal	Class	Journal name or ‘NA’.
2	Article	8	Article_Volume	Class	Journal volume or ‘NA’.
2	Article	9	Article_Page	Class	First and last journal pages (‘First journal page-Last journal page’) or ‘NA’.
2	Article	10	IDEQ_Equation	Index	Corresponding index from the ‘Literature_Search’ table. Secondary key of the ‘Article’ table.
3	Site	11	IDSite	Index	Index of each site from each article. Primary key of the ‘Site’ table.
3	Site	12	Site_Name	Class	Site name.
3	Site	13	Site_Country	Class	Site country.
3	Site	14	Site_City_State_Region	Class	City, state and/or region name(s) where the site is precisely located or the nearest located or ‘NA’.
3	Site	15	Site_Latitude	Numerical	Site latitude coordinate.
3	Site	16	Site_Longitude	Numerical	Site longitude coordinate.
3	Site	17	Site_Coordinate_Source	Class	Source of latitude and longitude coordinates. The source is from the article when latitude and longitude coordinates are originally reported or from the National Aeronautics and Space Administration (NASA) finder (http://mynasadata.larc.nasa.gov/latitudelongitude-finder) when coordinates are not originally reported.
3	Site	18	Site_Soil_Depth_Variable_m	Numerical	Soil depth layer (in m) at which soil attributes are determined or ‘NA’. When soil attributes are reported for many soil depth layers, soil attributes are averaged over many soil depth layers.
3	Site	19	Site_Soil_Classification_Name	Class	Soil classification name(s) or ‘NA’.
3	Site	20	Site_Soil_Texture_Name	Class	Soil texture name(s) or ‘NA’.
3	Site	21	Site_Soil_Sand_Percentage	Numerical	Soil average percentage of sand or ‘NA’. When many percentages of sand are reported for a given soil depth layer, all percentages of sand are added for the given soil depth layer.
3	Site	22	Site_Soil_Silt_Percentage	Numerical	Soil average percentage of silt or ‘NA’.
3	Site	23	Site_Soil_Clay_Percentage	Numerical	Soil average percentage of clay or ‘NA’.
3	Site	24	Site_Soil_pH	Numerical	Soil average pH or ‘NA’.
3	Site	25	Site_Soil_pH_Basis	Class	Soil chemical basis at which soil average pH ('Ca'/'CaCl_2_'/'H_2_O'/'KCl') is determined or ‘NA’.
3	Site	26	Site_Soil_Organic_Matter_Percentage	Numerical	Soil average percentage of organic matter or ‘NA’.
3	Site	27	Site_Soil_N_Percentage	Numerical	Soil average percentage of nitrogen (N) or ‘NA’.
3	Site	28	Site_Soil_N_Percentage_Type	Class	Type of soil average percentage of nitrogen ('Total'/'Organic') or ‘NA’. This attribute may be completed with soil nitrogen quantity or soil nitrogen percentage at seeding from the 'Crop_N_Soil_Quantity_Percentage_Seeding' attribute or at harvest from the 'Crop_N_Soil_Quantity_Percentage_Harvest' attribute.
3	Site	29	Site_Soil_N_Percentage	Numerical	Soil average percentage of nitrogen (N) or ‘NA’.
3	Site	29	Site_Soil_C_Percentage	Numerical	Soil average percentage of carbon (C) or ‘NA’.
3	Site	30	Site_Soil_C_Percentage_Type	Class	Type of soil average percentage of carbon ('Total'/'Organic') or ‘NA’.
3	Site	31	Site_Precipitation_mm	Numerical	Site average precipitation (in mm) or ‘NA’.
3	Site	32	Site_Precipitation_Period	Class	Period ('Annual'/'Growing season') at which site average precipitation is determined or ‘NA’.
3	Site	33	Site_Precipitation_Period_Month	Date	First and last calendar months of the period ('First month-Last month') at which site average precipitation is determined or ‘NA’. Months are abbreviated: 'Jan.': January, 'Feb.': February, 'Mar.': March, 'Apr.': April, 'May': May, 'Jun.': June, 'Jul.': July, 'Aug.': August, 'Sep.': September, 'Oct.': October, 'Nov.': November, 'Dec.': December.
3	Site	34	Site_Precipitation_Period_Year	Date	First and last calendar years of the period ('First year-Last year') at which site average precipitation is determined or ‘NA’.
3	Site	35	Site_Temperature_Celsius	Numerical	Site average temperature (in Celsius) or ‘NA’.
3	Site	36	Site_Temperature_Period	Class	Period ('Annual'/'Growing season') at which site average temperature is determined or ‘NA’.
3	Site	37	Site_Temperature_Period_Month	Date	First and last calendar months of the period ('First month-Last month') at which site average temperature is determined or ‘NA’. Months are abbreviated: 'Jan.': January, 'Feb.': February, 'Mar.': March, 'Apr.': April, 'May': May, 'Jun.': June, 'Jul.': July, 'Aug.': August, 'Sep.': September, 'Oct.': October, 'Nov.': November, 'Dec.': December.
3	Site	38	Site_Temperature_Period_Year	Date	First and last calendar years of the period ('First year-Last year') at which site average temperature is determined or ‘NA’.
3	Site	39	identifiant_Paper	Index	Corresponding index from the 'Article' table. Secondary key of the 'Site' table.
4	Crop_Sequence_Trt	40	IDRotation	Index	Index of each crop sequence and/or treatment from each site. Primary key of the 'Crop_Sequence_Trt' table.
4	Crop_Sequence_Trt	41	Crop_Sequence_Trt_Name	Class	Crop sequence and/or treatment name(s). Common names of the 'Crop_Species_Common_Name' attribute from the 'Crop' table are reported. See Data Records section for further information.
4	Crop_Sequence_Trt	42	Crop_Sequence_Trt_Species_Order	Class	Species order. Common names of the 'Crop_Species_Common_Name' attribute from the 'Crop' table are reported. Each common name is separated by a '-'. See Data Records section for further information.
4	Crop_Sequence_Trt	43	Crop_Sequence_Trt_Species_Number	Numerical	Species number. Monoculture accounts for one species. Fallow accounts for zero species.
4	Crop_Sequence_Trt	44	Crop_Sequence_Trt_Species_Legume_Harvested	Binary	There is ('1') or there is not ('0') at least one harvested legume species in the crop sequence. Fallow is reported as a non-legume species.
4	Crop_Sequence_Trt	45	Crop_Sequence_Trt_Cultivar_Name	Class	Cultivar name(s) of each species in the crop sequence or ‘NA’. Cultivar names of preceding and following species in the crop sequence are separated by a '-'. ‘NA’ is reported for fallow.
4	Crop_Sequence_Trt	46	Crop_Sequence_Trt_Growing_Season_Number	Numerical	Number of consecutive growing season(s) in the crop sequence.
4	Crop_Sequence_Trt	47	IDSite_Site	Index	Corresponding index from the 'Site' table. Secondary key of the 'Crop_Sequence_Trt' table.
5	Crop	48	IDCrop	Index	Index of each crop from each crop sequence and/or treatment. Primary key of the 'Crop' table.
5	Crop	49	Crop_Sequence_Treatment_Name	Class	Crop sequence and/or treatment name(s). Common names of the 'Crop_Species_Common_Name' attribute from the 'Crop' table are reported. See Data Records section for further information.
5	Crop	50	Crop_Site_Growing_Season_ID	Index	Index for each crop grown at the same field site during the same growing seasons.
5	Crop	51	Crop_Growing_Season_Year_First	Date	First calendar year at which the crop is seeded and/or the growing season starts or ‘NA’. When values are averaged over many growing seasons, only the calendar year of the first growing season is reported. For instance, if values are averaged over 5 growing seasons from 2005 to 2010, then only 2005 is reported.
5	Crop	52	Crop_Growing_Season_Year_Last	Date	Last calendar year at which the crop is harvested and/or the growing season ends or ‘NA’. When values are averaged over many growing seasons, only the calendar year of the last growing season is reported. For instance, if values are averaged over 5 growing seasons from 2005 to 2010, then only 2010 is reported.
5	Crop	53	Crop_Growing_Season_Number	Numerical	Number of growing season(s). When values are averaged over many growing seasons, the number of growing seasons is reported. For instance, if values are averaged from 2005 to 2010, then 5 growing seasons are reported.
5	Crop	54	Crop_Species_Scientific_Name	Class	Species scientific name. See Data Records section for further information.
5	Crop	55	Crop_Species_Common_Name	Class	Species common name. See Data Records section for further information.
5	Crop	56	Crop_Species_Legume	Binary	The species is ('1') or is not ('0') a legume species. Fallow is reported as a non-legume species.
5	Crop	57	Crop_Date_Seeding	Date	Average seeding date ('Day Month Year') or 'NA NA NA'. When values are averaged over many growing seasons, seeding dates for each growing season are reported. Months are abbreviated: 'Jan.': January, 'Feb.': February, 'Mar.': March, 'Apr.': April, 'May': May, 'Jun.': June, 'Jul.': July, 'Aug.': August, 'Sep.': September, 'Oct.': October, 'Nov.': November, 'Dec.': December.
5	Crop	58	Crop_Date_Harvest	Date	Average harvest date ('Day Month Year') or 'NA NA NA'. When values are averaged over many growing seasons, harvest dates for each growing season are reported. Months are abbreviated: 'Jan.': January, 'Feb.': February, 'Mar.': March, 'Apr.': April, 'May': May, 'Jun.': June, 'Jul.': July, 'Aug.': August, 'Sep.': September, 'Oct.': October, 'Nov.': November, 'Dec.': December.
5	Crop	59	Crop_Date_From_Seeding_To_Harvest_Day_Number	Numerical	Average number of Julian days from seeding to harvest dates or ‘NA’. See Data Records section for further information.
5	Crop	60	Crop_Following_Number	Binary	The species is ('1') or is not ('0') a following species. See Data Records section for further information.
5	Crop	61	Crop_Multiple_Following_For_Same_Preceding	Binary	The row is ('1') or is not ('0') a duplicated row when values are averaged over a same crop preceding different crops. See Data Records section for further information.
5	Crop	62	Crop_Across_Treatment_Averaged_Value	Binary	The values are ('1') or are not ('0') averaged over many treatments. See Data Records section for further information.
5	Crop	63	Crop_Across_Treatment_Averaged_Value_Type	Class	If values are averaged over many treatments, then the type of treatment(s) is reported. If values are not averaged over many treatments, 'NULL' is reported.
5	Crop	64	Crop_Across_Species_Same_Treatment_Value	Binary	The species shares ('1') or does not share ('0') the same treatment(s) tested on other species grown at the same field site during the same growing seasons. See Data Records section for further information.
5	Crop	65	Crop_Across_Species_Same_Treatment_Value_Type	Class	If the species does not share the same treatment(s) tested on other species grown at the same field site during the same growing seasons, then the type of different treatments is reported. If the species shares the same treatment(s) tested on other species grown at the same field site during the same growing seasons, then 'NULL' is reported. See Data Records section for further information.
5	Crop	66	Crop_Replicate_Number	Numerical	Number of replicates or ‘NA’. See Data Records section for further information.
5	Crop	67	Crop_Yield_Grain	Numerical	Grain yield or ‘NA’. Shells are included but pods are not, except for *Arachis hypogaea* (peanut). See Data Records section for further information.
5	Crop	68	Crop_Yield_Grain_Unit	Class	Unit of grain yield or ‘NA’.
5	Crop	69	Crop_Yield_Grain_Error	Numerical	Error term of grain yield or ‘NA’.
5	Crop	70	Crop_Yield_Grain_Error_Type	Class	Error type of grain yield or ‘NA’. Error types are abbreviated: 'CD0.05': Confidence Distribution at the probability level of 5%, 'CV': Coefficient of Variation (%), 'DMR0.05': Duncan's Multiple Range Test at the probability level of 5%, 'LSD0.01': Fisher's Least Significant Difference Test at the probability level of 1%, 'LSD0.05': Fisher's Least Significant Difference Test at the probability level of 5%, 'LSD0.10': Fisher's Least Significant Difference Test at the probability level of 10%, 's.d.': Standard Deviation, ‘s.e.’: Standard Error, 'SED': Standard Error of the Difference, 'SEDM': Standard Error of the Difference between Means, ‘s.e.m.’: Standard Error of the Mean, 'HSD0.05': Tukey's Honest Significant Difference Test at the probability level of 5%, 'HSD0.10': Tukey's Honest Significant Difference Test at the probability level of 10%.
5	Crop	71	Crop_Yield_Grain_DM_Percentage	Numerical	Dry matter percentage of grain yield or ‘NA’.
5	Crop	72	Crop_Biomass_Aerial	Numerical	Aerial biomass or ‘NA’.
5	Crop	73	Crop_Biomass_Aerial_Unit	Class	Unit of aerial biomass or ‘NA’.
5	Crop	74	Crop_Biomass_Aerial_Error	Numerical	Error term of aerial biomass or ‘NA’.
5	Crop	75	Crop_Biomass_Aerial_Error_Type	Class	Error type of aerial biomass or ‘NA’. Error types are abbreviated: 'CD0.05': Confidence Distribution at the probability level of 5%, 'CV': Coefficient of Variation (%), 'DMR0.05': Duncan's Multiple Range Test at the probability level of 5%, 'LSD0.01': Fisher's Least Significant Difference Test at the probability level of 1%, 'LSD0.05': Fisher's Least Significant Difference Test at the probability level of 5%, 'LSD0.10': Fisher's Least Significant Difference Test at the probability level of 10%, 's.d.': Standard Deviation, ‘s.e.’: Standard Error, 'SED': Standard Error of the Difference, 'SEDM': Standard Error of the Difference between Means, ‘s.e.m.’: Standard Error of the Mean, 'HSD0.05': Tukey's Honest Significant Difference Test at the probability level of 5%, 'HSD0.10': Tukey's Honest Significant Difference Test at the probability level of 10%.
5	Crop	76	Crop_Biomass_Aerial_DM_Percentage	Numerical	Dry matter percentage of aerial biomass or ‘NA’.
5	Crop	77	Crop_Biomass_Aerial_Definition	Class	Definition of aerial components in aerial biomass or ‘NA’.
5	Crop	78	Crop_Biomass_Aerial_Stage_Detailed	Date	Detailed phenology stage (i.e., originally stated in the article) at which aerial biomass is determined or ‘NA’.
5	Crop	79	Crop_Biomass_Aerial_Stage_Simplified	Date	Simplified phenology stage ('Before physiological maturity'/'Physiological maturity') at which aerial biomass is determined or ‘NA’.
5	Crop	80	Crop_Harvest_Index	Numerical	Harvest index or ‘NA’. See Data Records section for further information.
5	Crop	81	Crop_Harvest_Index_Error	Numerical	Error term of harvest index or ‘NA’.
5	Crop	82	Crop_Harvest_Index_Error_Type	Class	Error type of harvest index or ‘NA’. Error types are abbreviated: 'CD0.05': Confidence Distribution at the probability level of 5%, 'CV': Coefficient of Variation (%), 'DMR0.05': Duncan's Multiple Range Test at the probability level of 5%, 'LSD0.01': Fisher's Least Significant Difference Test at the probability level of 1%, 'LSD0.05': Fisher's Least Significant Difference Test at the probability level of 5%, 'LSD0.10': Fisher's Least Significant Difference Test at the probability level of 10%, 's.d.': Standard Deviation, ‘s.e.’: Standard Error, 'SED': Standard Error of the Difference, 'SEDM': Standard Error of the Difference between Means, ‘s.e.m.’: Standard Error of the Mean, 'HSD0.05': Tukey's Honest Significant Difference Test at the probability level of 5%, 'HSD0.10': Tukey's Honest Significant Difference Test at the probability level of 10%.
5	Crop	83	Crop_N_Quantity_Grain	Numerical	Grain nitrogen quantity or ‘NA’.
5	Crop	84	Crop_N_Quantity_Grain_Unit	Class	Unit of grain nitrogen quantity or ‘NA’.
5	Crop	85	Crop_N_Quantity_Grain_Error	Numerical	Error term of grain nitrogen quantity or ‘NA’.
5	Crop	86	Crop_N_Quantity_Grain_Error_Type	Class	Error type of grain nitrogen quantity or ‘NA’. Error types are abbreviated: 'CD0.05': Confidence Distribution at the probability level of 5%, 'CV': Coefficient of Variation (%), 'DMR0.05': Duncan's Multiple Range Test at the probability level of 5%, 'LSD0.01': Fisher's Least Significant Difference Test at the probability level of 1%, 'LSD0.05': Fisher's Least Significant Difference Test at the probability level of 5%, 'LSD0.10': Fisher's Least Significant Difference Test at the probability level of 10%, 's.d.': Standard Deviation, ‘s.e.’: Standard Error, 'SED': Standard Error of the Difference, 'SEDM': Standard Error of the Difference between Means, ‘s.e.m.’: Standard Error of the Mean, 'HSD0.05': Tukey's Honest Significant Difference Test at the probability level of 5%, 'HSD0.10': Tukey's Honest Significant Difference Test at the probability level of 10%.
5	Crop	87	Crop_N_Quantity_Aerial	Numerical	Aerial nitrogen quantity or ‘NA’.
5	Crop	88	Crop_N_Quantity_Aerial_Unit	Class	Unit of aerial nitrogen quantity or ‘NA’.
5	Crop	89	Crop_N_Quantity_Aerial_Error	Numerical	Error term of aerial nitrogen quantity or ‘NA’.
5	Crop	90	Crop_N_Quantity_Aerial_Error_Type	Class	Error type of aerial nitrogen quantity or ‘NA’. Error types are abbreviated: 'CD0.05': Confidence Distribution at the probability level of 5%, 'CV': Coefficient of Variation (%), 'DMR0.05': Duncan's Multiple Range Test at the probability level of 5%, 'LSD0.01': Fisher's Least Significant Difference Test at the probability level of 1%, 'LSD0.05': Fisher's Least Significant Difference Test at the probability level of 5%, 'LSD0.10': Fisher's Least Significant Difference Test at the probability level of 10%, ‘s.d.’: Standard Deviation, ‘s.e.’: Standard Error, 'SED': Standard Error of the Difference, 'SEDM': Standard Error of the Difference between Means, ‘s.e.m.’: Standard Error of the Mean, 'HSD0.05': Tukey's Honest Significant Difference Test at the probability level of 5%, 'HSD0.10': Tukey's Honest Significant Difference Test at the probability level of 10%.
5	Crop	91	Crop_N_Quantity_Aerial_Definition	Class	Definition of aerial components in aerial nitrogen quantity or ‘NA’.
5	Crop	92	Crop_N_Percentage_Grain	Numerical	Grain nitrogen percentage or ‘NA’. See Data Records section for further information.
5	Crop	93	Crop_N_Percentage_Grain_Error	Numerical	Error term of grain nitrogen percentage or ‘NA’.
5	Crop	94	Crop_N_Percentage_Grain_Error_Type	Class	Error type of grain nitrogen percentage or ‘NA’. Error types are abbreviated: 'CD0.05': Confidence Distribution at the probability level of 5%, 'CV': Coefficient of Variation (%), 'DMR0.05': Duncan's Multiple Range Test at the probability level of 5%, 'LSD0.01': Fisher's Least Significant Difference Test at the probability level of 1%, 'LSD0.05': Fisher's Least Significant Difference Test at the probability level of 5%, 'LSD0.10': Fisher's Least Significant Difference Test at the probability level of 10%, ‘s.d.’: Standard Deviation, ‘s.e.’: Standard Error, 'SED': Standard Error of the Difference, 'SEDM': Standard Error of the Difference between Means, ‘s.e.m.’: Standard Error of the Mean, 'HSD0.05': Tukey's Honest Significant Difference Test at the probability level of 5%, 'HSD0.10': Tukey's Honest Significant Difference Test at the probability level of 10%.
5	Crop	95	Crop_N_Percentage_Aerial	Numerical	Aerial nitrogen percentage or ‘NA’. See Data Records section for further information.
5	Crop	96	Crop_N_Percentage_Aerial_Error	Numerical	Error term of aerial nitrogen percentage or ‘NA’.
5	Crop	97	Crop_N_Percentage_Aerial_Error_Type	Class	Error type of aerial nitrogen percentage or ‘NA’. Error types are abbreviated: 'CD0.05': Confidence Distribution at the probability level of 5%, 'CV': Coefficient of Variation (%), 'DMR0.05': Duncan's Multiple Range Test at the probability level of 5%, 'LSD0.01': Fisher's Least Significant Difference Test at the probability level of 1%, 'LSD0.05': Fisher's Least Significant Difference Test at the probability level of 5%, 'LSD0.10': Fisher's Least Significant Difference Test at the probability level of 10%, 's.d.': Standard Deviation, ‘s.e.’: Standard Error, 'SED': Standard Error of the Difference, 'SEDM': Standard Error of the Difference between Means, ‘s.e.m.’: Standard Error of the Mean, 'HSD0.05': Tukey's Honest Significant Difference Test at the probability level of 5%, 'HSD0.10': Tukey's Honest Significant Difference Test at the probability level of 10%.
5	Crop	98	Crop_N_Percentage_Aerial_Definition	Class	Definition of aerial components in aerial nitrogen percentage or ‘NA’.
5	Crop	99	Crop_N_Harvest_Index	Numerical	Nitrogen harvest index or ‘NA’. See Data Records section for further information.
5	Crop	100	Crop_N_Harvest_Index_Error	Numerical	Error term of nitrogen harvest index or ‘NA’.
5	Crop	101	Crop_N_Harvest_Index_Error_Type	Class	Error type of nitrogen harvest index or ‘NA’. Error types are abbreviated: 'CD0.05': Confidence Distribution at the probability level of 5%, 'CV': Coefficient of Variation (%), 'DMR0.05': Duncan's Multiple Range Test at the probability level of 5%, 'LSD0.01': Fisher's Least Significant Difference Test at the probability level of 1%, 'LSD0.05': Fisher's Least Significant Difference Test at the probability level of 5%, 'LSD0.10': Fisher's Least Significant Difference Test at the probability level of 10%, 's.d.': Standard Deviation, ‘s.e.’: Standard Error, 'SED': Standard Error of the Difference, 'SEDM': Standard Error of the Difference between Means, ‘s.e.m.’: Standard Error of the Mean, 'HSD0.05': Tukey's Honest Significant Difference Test at the probability level of 5%, 'HSD0.10': Tukey's Honest Significant Difference Test at the probability level of 10%.
5	Crop	102	Crop_N_Fixed_Quantity_Aerial	Numerical	Aerial fixed nitrogen quantity or ‘NA’. See Data Records section for further information.
5	Crop	103	Crop_N_Fixed_Quantity_Aerial_Unit	Class	Unit of aerial fixed nitrogen quantity or ‘NA’.
5	Crop	104	Crop_N_Fixed_Quantity_Aerial_Error	Numerical	Error term of aerial fixed nitrogen quantity or ‘NA’.
5	Crop	105	Crop_N_Fixed_Quantity_Aerial_Error_Type	Class	Error type of aerial fixed nitrogen quantity or ‘NA’. Error types are abbreviated: 'CD0.05': Confidence Distribution at the probability level of 5%, 'CV': Coefficient of Variation (%), 'DMR0.05': Duncan's Multiple Range Test at the probability level of 5%, 'LSD0.01': Fisher's Least Significant Difference Test at the probability level of 1%, 'LSD0.05': Fisher's Least Significant Difference Test at the probability level of 5%, 'LSD0.10': Fisher's Least Significant Difference Test at the probability level of 10%, 's.d.': Standard Deviation, ‘s.e.’: Standard Error, 'SED': Standard Error of the Difference, 'SEDM': Standard Error of the Difference between Means, ‘s.e.m.’: Standard Error of the Mean, 'HSD0.05': Tukey's Honest Significant Difference Test at the probability level of 5%, 'HSD0.10': Tukey's Honest Significant Difference Test at the probability level of 10%.
5	Crop	106	Crop_N_Fixed_Quantity_Aerial_Definition	Class	Definition of aerial components in aerial fixed nitrogen quantity or ‘NA’.
5	Crop	107	Crop_N_Fixed_Percentage_Aerial	Numerical	Aerial fixed nitrogen percentage or ‘NA’.
5	Crop	108	Crop_N_Fixed_Percentage_Aerial_Error	Numerical	Error term of aerial fixed nitrogen percentage or ‘NA’.
5	Crop	109	Crop_N_Fixed_Percentage_Aerial_Error_Type	Class	Error type of aerial fixed nitrogen percentage or ‘NA’. Error types are abbreviated: 'CD0.05': Confidence Distribution at the probability level of 5%, 'CV': Coefficient of Variation (%), 'DMR0.05': Duncan's Multiple Range Test at the probability level of 5%, 'LSD0.01': Fisher's Least Significant Difference Test at the probability level of 1%, 'LSD0.05': Fisher's Least Significant Difference Test at the probability level of 5%, 'LSD0.10': Fisher's Least Significant Difference Test at the probability level of 10%, 's.d.': Standard Deviation, ‘s.e.’: Standard Error, 'SED': Standard Error of the Difference, 'SEDM': Standard Error of the Difference between Means, ‘s.e.m.’: Standard Error of the Mean, 'HSD0.05': Tukey's Honest Significant Difference Test at the probability level of 5%, 'HSD0.10': Tukey's Honest Significant Difference Test at the probability level of 10%.
5	Crop	110	Crop_N_Fixed_Percentage_Aerial_Method	Class	Method at which aerial fixed nitrogen percentage is determined or ‘NA’.
5	Crop	111	Crop_N_Fixed_Percentage_Aerial_Reference_Species	Class	Species scientific name(s) of non-fixing reference species at which aerial fixed nitrogen percentage is determined or ‘NA’.
5	Crop	112	Crop_N_Fixed_Percentage_Aerial_Stage_Detailed	Date	Detailed phenology stage (i.e., originally stated in the article) at which aerial fixed nitrogen percentage is determined or ‘NA’.
5	Crop	113	Crop_N_Fixed_Percentage_Aerial_Stage_Simplified	Date	Simplified phenology stage ('Before physiological maturity'/'Physiological maturity') at which aerial fixed nitrogen percentage is determined or ‘NA’.
5	Crop	114	Crop_Protein_Quantity_Percentage_Grain	Numerical	Grain protein quantity or grain protein percentage or ‘NA’.
5	Crop	115	Crop_Protein_Quantity_Percentage_Grain_Unit	Class	Unit of grain protein quantity or grain protein percentage or ‘NA’.
5	Crop	116	Crop_Protein_Quantity_Percentage_Grain_Error	Numerical	Error term of grain protein quantity or grain protein percentage or ‘NA’.
5	Crop	117	Crop_Protein_Quantity_Percentage_Grain_Error_Type	Class	Error type of grain protein quantity or grain protein percentage or ‘NA’. Error types are abbreviated: 'CD0.05': Confidence Distribution at the probability level of 5%, 'CV': Coefficient of Variation (%), 'DMR0.05': Duncan's Multiple Range Test at the probability level of 5%, 'LSD0.01': Fisher's Least Significant Difference Test at the probability level of 1%, 'LSD0.05': Fisher's Least Significant Difference Test at the probability level of 5%, 'LSD0.10': Fisher's Least Significant Difference Test at the probability level of 10%, 's.d.': Standard Deviation, ‘s.e.’: Standard Error, 'SED': Standard Error of the Difference, 'SEDM': Standard Error of the Difference between Means, ‘s.e.m.’: Standard Error of the Mean, 'HSD0.05': Tukey's Honest Significant Difference Test at the probability level of 5%, 'HSD0.10': Tukey's Honest Significant Difference Test at the probability level of 10%.
5	Crop	118	Crop_N_Balance_Simplified	Numerical	Simplified nitrogen balance or ‘NA’. See Data Records section for further information.
5	Crop	119	Crop_N_Balance_Simplified_Unit	Class	Unit of simplified nitrogen balance or ‘NA’. Simplified nitrogen balance is only reported in nitrogen.
5	Crop	120	Crop_N_Balance_Simplified_Error	Numerical	Error term of simplified nitrogen balance or ‘NA’.
5	Crop	121	Crop_N_Balance_Simplified_Error_Type	Class	Error type of simplified nitrogen balance or ‘NA’. Error types are abbreviated: 'CD0.05': Confidence Distribution at the probability level of 5%, 'CV': Coefficient of Variation (%), 'DMR0.05': Duncan's Multiple Range Test at the probability level of 5%, 'LSD0.01': Fisher's Least Significant Difference Test at the probability level of 1%, 'LSD0.05': Fisher's Least Significant Difference Test at the probability level of 5%, 'LSD0.10': Fisher's Least Significant Difference Test at the probability level of 10%, 's.d.': Standard Deviation, ‘s.e.’: Standard Error, 'SED': Standard Error of the Difference, 'SEDM': Standard Error of the Difference between Means, ‘s.e.m.’: Standard Error of the Mean, 'HSD0.05': Tukey's Honest Significant Difference Test at the probability level of 5%, 'HSD0.10': Tukey's Honest Significant Difference Test at the probability level of 10%.
5	Crop	122	Crop_N_Balance_Simplified_Equation	Class	Equation of simplified nitrogen balance or ‘NA’. See Data Records section for further information.
5	Crop	123	Crop_N_Soil_Quantity_Percentage_Seeding	Numerical	Soil nitrogen quantity or soil nitrogen percentage at seeding or ‘NA’.
5	Crop	124	Crop_N_Soil_Quantity_Percentage_Seeding_Unit	Class	Unit of soil nitrogen quantity or soil nitrogen percentage at seeding or ‘NA’.
5	Crop	125	Crop_N_Soil_Quantity_Percentage_Seeding_Type	Class	Type of soil nitrogen quantity or soil nitrogen percentage at seeding ('Mineral'/'Nitrate'/'Nitrogen') or ‘NA’. Mineral nitrogen is defined as ammonium plus nitrate.
5	Crop	126	Crop_N_Soil_Quantity_Percentage_Seeding_Error	Numerical	Error term of soil nitrogen quantity or soil nitrogen percentage at seeding or ‘NA’.
5	Crop	127	Crop_N_Soil_Quantity_Percentage_Seeding_Error_Type	Class	Error type of soil nitrogen quantity or soil nitrogen percentage at seeding or ‘NA’. Error types are abbreviated: 'CD0.05': Confidence Distribution at the probability level of 5%, 'CV': Coefficient of Variation (%), 'DMR0.05': Duncan's Multiple Range Test at the probability level of 5%, 'LSD0.01': Fisher's Least Significant Difference Test at the probability level of 1%, 'LSD0.05': Fisher's Least Significant Difference Test at the probability level of 5%, 'LSD0.10': Fisher's Least Significant Difference Test at the probability level of 10%, ‘s.d.’: Standard Deviation, ‘s.e.’: Standard Error, 'SED': Standard Error of the Difference, 'SEDM': Standard Error of the Difference between Means, ‘s.e.m.’: Standard Error of the Mean, 'HSD0.05': Tukey's Honest Significant Difference Test at the probability level of 5%, 'HSD0.10': Tukey's Honest Significant Difference Test at the probability level of 10%.
5	Crop	128	Crop_N_Soil_Quantity_Percentage_Seeding_Depth	Numerical	Soil depth layer at which soil nitrogen quantity or soil nitrogen percentage at seeding is determined or ‘NA’.
5	Crop	129	Crop_N_Soil_Quantity_Percentage_Seeding_Depth_Unit	Class	Unit of soil depth layer at which soil nitrogen quantity or soil nitrogen percentage at seeding is determined or ‘NA’.
5	Crop	130	Crop_N_Soil_Quantity_Percentage_Seeding_Date	Date	Date at which soil nitrogen quantity or soil nitrogen percentage at seeding is determined or ‘NA’.
5	Crop	131	Crop_N_Soil_Quantity_Percentage_Harvest	Numerical	Soil nitrogen quantity or soil nitrogen percentage at harvest or ‘NA’.
5	Crop	132	Crop_N_Soil_Quantity_Percentage_Harvest_Unit	Class	Unit of soil nitrogen quantity or soil nitrogen percentage at harvest or ‘NA’.
5	Crop	133	Crop_N_Soil_Quantity_Percentage_Harvest_Type	Class	Type of soil nitrogen quantity or soil nitrogen percentage at harvest ('Mineral'/'Nitrate'/'Nitrogen') or ‘NA’. Mineral nitrogen is defined as ammonium plus nitrate.
5	Crop	134	Crop_N_Soil_Quantity_Percentage_Harvest_Error	Numerical	Error term of soil nitrogen quantity or soil nitrogen percentage at harvest or ‘NA’.
5	Crop	135	Crop_N_Soil_Quantity_Percentage_Harvest_Error_Type	Class	Error type of soil nitrogen quantity or soil nitrogen percentage at harvest or ‘NA’. Error types are abbreviated: 'CD0.05': Confidence Distribution at the probability level of 5%, 'CV': Coefficient of Variation (%), 'DMR0.05': Duncan's Multiple Range Test at the probability level of 5%, 'LSD0.01': Fisher's Least Significant Difference Test at the probability level of 1%, 'LSD0.05': Fisher's Least Significant Difference Test at the probability level of 5%, 'LSD0.10': Fisher's Least Significant Difference Test at the probability level of 10%, ‘s.d.’: Standard Deviation, ‘s.e.’: Standard Error, 'SED': Standard Error of the Difference, 'SEDM': Standard Error of the Difference between Means, ‘s.e.m.’: Standard Error of the Mean, 'HSD0.05': Tukey's Honest Significant Difference Test at the probability level of 5%, 'HSD0.10': Tukey's Honest Significant Difference Test at the probability level of 10%.
5	Crop	136	Crop_N_Soil_Quantity_Percentage_Harvest_Depth	Numerical	Soil depth layer at which soil nitrogen quantity or soil nitrogen percentage at harvest is determined or ‘NA’.
5	Crop	137	Crop_N_Soil_Quantity_Percentage_Harvest_Depth_Unit	Class	Unit of soil depth layer at which soil nitrogen quantity or soil nitrogen percentage at harvest is determined or ‘NA’.
5	Crop	138	Crop_N_Soil_Quantity_Percentage_Harvest_Date	Date	Date at which soil nitrogen quantity or soil nitrogen percentage at harvest is determined or ‘NA’.
5	Crop	139	Crop_Water_Use_Balance	Numerical	Water use or water balance or ‘NA’.
5	Crop	140	Crop_Water_Use_Balance_Unit	Class	Unit of water use or water balance or ‘NA’.
5	Crop	141	Crop_Water_Use_Balance_Error	Numerical	Error term of water use or water balance or ‘NA’.
5	Crop	142	Crop_Water_Use_Balance_Error_Type	Class	Error type of water use or water balance or ‘NA’. Error types are abbreviated: 'CD0.05': Confidence Distribution at the probability level of 5%, 'CV': Coefficient of Variation (%), 'DMR0.05': Duncan's Multiple Range Test at the probability level of 5%, 'LSD0.01': Fisher's Least Significant Difference Test at the probability level of 1%, 'LSD0.05': Fisher's Least Significant Difference Test at the probability level of 5%, 'LSD0.10': Fisher's Least Significant Difference Test at the probability level of 10%, ‘s.d.’: Standard Deviation, ‘s.e.’: Standard Error, 'SED': Standard Error of the Difference, 'SEDM': Standard Error of the Difference between Means, ‘s.e.m.’: Standard Error of the Mean, 'HSD0.05': Tukey's Honest Significant Difference Test at the probability level of 5%, 'HSD0.10': Tukey's Honest Significant Difference Test at the probability level of 10%.
5	Crop	143	Crop_Water_Use_Balance_Equation	Class	Equation of water use or water balance or ‘NA’. See Data Records section for further information.
5	Crop	144	Crop_Water_Use_Balance_Efficiency_Grain	Numerical	Grain water use efficiency or grain water balance efficiency or ‘NA’. See Data Records section for further information.
5	Crop	145	Crop_Water_Use_Balance_Efficiency_Grain_Unit	Class	Unit of grain water use efficiency or grain water balance efficiency or ‘NA’.
5	Crop	146	Crop_Water_Use_Balance_Efficiency_Grain_Error	Numerical	Error term of grain water use efficiency or grain water balance efficiency or ‘NA’.
5	Crop	147	Crop_Water_Use_Balance_Efficiency_Grain_Error_Type	Class	Error type of grain water use efficiency or grain water balance efficiency or ‘NA’. Error types are abbreviated: 'CD0.05': Confidence Distribution at the probability level of 5%, 'CV': Coefficient of Variation (%), 'DMR0.05': Duncan's Multiple Range Test at the probability level of 5%, 'LSD0.01': Fisher's Least Significant Difference Test at the probability level of 1%, 'LSD0.05': Fisher's Least Significant Difference Test at the probability level of 5%, 'LSD0.10': Fisher's Least Significant Difference Test at the probability level of 10%, ‘s.d.’: Standard Deviation, ‘s.e.’: Standard Error, 'SED': Standard Error of the Difference, 'SEDM': Standard Error of the Difference between Means, ‘s.e.m.’: Standard Error of the Mean, 'HSD0.05': Tukey's Honest Significant Difference Test at the probability level of 5%, 'HSD0.10': Tukey's Honest Significant Difference Test at the probability level of 10%.
5	Crop	148	Crop_Water_Use_Balance_Efficiency_Aerial	Numerical	Aerial water use efficiency or aerial water balance efficiency or ‘NA’. See Data Records section for further information.
5	Crop	149	Crop_Water_Use_Balance_Efficiency_Aerial_Unit	Class	Unit of aerial water use efficiency or aerial water balance efficiency or ‘NA’.
5	Crop	150	Crop_Water_Use_Balance_Efficiency_Aerial_Error	Numerical	Error term of aerial water use efficiency or aerial water balance efficiency or ‘NA’.
5	Crop	151	Crop_Water_Use_Balance_Efficiency_Aerial_Error_Type	Class	Error type of aerial water use efficiency or aerial water balance efficiency or ‘NA’. Error types are abbreviated: 'CD0.05': Confidence Distribution at the probability level of 5%, 'CV': Coefficient of Variation (%), 'DMR0.05': Duncan's Multiple Range Test at the probability level of 5%, 'LSD0.01': Fisher's Least Significant Difference Test at the probability level of 1%, 'LSD0.05': Fisher's Least Significant Difference Test at the probability level of 5%, 'LSD0.10': Fisher's Least Significant Difference Test at the probability level of 10%, ‘s.d.’: Standard Deviation, ‘s.e.’: Standard Error, 'SED': Standard Error of the Difference, 'SEDM': Standard Error of the Difference between Means, ‘s.e.m.’: Standard Error of the Mean, 'HSD0.05': Tukey's Honest Significant Difference Test at the probability level of 5%, 'HSD0.10': Tukey's Honest Significant Difference Test at the probability level of 10%.
5	Crop	152	Crop_Water_Use_Balance_Efficiency_Aerial_Definition	Class	Definition of aerial components in aerial water use efficiency or aerial water balance efficiency or ‘NA’.
5	Crop	153	IDRotation_CropSystem	Index	Corresponding index from the 'Crop_Sequence' table. Secondary key of the 'Crop' table.
6	Tillage	154	IDTillage	Index	Index of each tillage management from each crop. Primary key of the 'Tillage' table.
6	Tillage	155	Tillage_Presence_Tillage	Binary	There is ('1') or there is not ('0') tillage management or ‘NA’. When the crop is 'seeded directly', then '0' is reported in the 'Tillage_Presence_Tillage' attribute, and '0.00' and 'm' are reported in the 'Tillage_Presence_Tillage_Depth' and 'Tillage_Presence_Tillage_Depth_Unit' attributes, respectively.
6	Tillage	156	Tillage_Presence_Tillage_Tool	Class	Tillage management tool(s) or ‘NA’.
6	Tillage	157	Tillage_Presence_Tillage_Depth	Numerical	If there is tillage management, then the tillage depth is reported. If there is no tillage management, 'NULL' is reported. Elsewhere, ‘NA’ is reported.
6	Tillage	158	Tillage_Presence_Tillage_Depth_Unit	Class	If there is tillage management, then the unit of tillage depth is reported. If there is no tillage management, 'NULL' is reported. Elsewhere, ‘NA’ is reported.
6	Tillage	159	Tillage_Incorporation_Preceding_Residue	Binary	There is ('1') or there is no ('0') incorporation of the preceding crop residues in soil. Elsewhere, ‘NA’ is reported. Incorporation of the preceding crop residues in soil is independently reported from the presence or the absence of tillage management. Except when the crop is 'seeded directly' or the preceding crop residues are 'burned', then '0' is reported in the 'Tillage_Incorporation_Preceding_Residue' attribute.
6	Tillage	160	Tillage_Preceding_Species_Scientific_Name	Class	Species scientific name of the preceding crop or ‘NA’.
6	Tillage	161	Tillage_Seeding_Depth	Numerical	Soil seeding depth or ‘NA’.
6	Tillage	162	Tillage_Seeding_Depth_Unit	Class	Unit of soil seeding depth or ‘NA’.
6	Tillage	163	Tillage_Seeding_Delay_Day	Binary	There is ('1') or there is not ('0') seeding delay or ‘NA’.
6	Tillage	164	Tillage_Seeding_Delay_Day_Number	Numerical	If there is seeding delay, then the number of seeding delayed days is reported. Elsewhere, ‘NA’ is reported.
6	Tillage	165	Tillage_Seeding_Row_Inter	Numerical	Inter-row spacing at seeding or ‘NA’.
6	Tillage	166	Tillage_Seeding_Row_Inter_Unit	Class	Unit of inter-row spacing at seeding or ‘NA’.
6	Tillage	167	Tillage_Seeding_Row_Intra	Numerical	Intra-row spacing at seeding or ‘NA’.
6	Tillage	168	Tillage_Seeding_Row_Intra_Unit	Class	Unit of intra-row spacing at seeding or ‘NA’.
6	Tillage	169	Tillage_Seeding_Density	Numerical	Seeding density or ‘NA’. Initial seeding density is reported but plant density after seeding is not.
6	Tillage	170	Tillage_Seeding_Density_Unit	Class	Unit of seeding density or ‘NA’.
6	Tillage	171	Tillage_Seeding_Inoculation	Binary	There is ('1') or there is not ('0') inoculation of legume species at seeding or ‘NA’. ‘NA’ is reported for non-legume species.
6	Tillage	172	IDCrop_Crop	Index	Corresponding index from the 'Crop' table. Secondary key of the 'Tillage' table.
7	Fertilization	173	IDFertilization	Index	Index of each fertilization management from each crop. Primary key of the 'Fertilization' table.
7	Fertilization	174	Fertilization_NPK	Class	Fertilization nutrient (Nitrogen ('N')/Phosphate ('P')/Potassium ('K')) or ‘NA’.
7	Fertilization	175	Fertilization_NPK_Dose	Numerical	Fertilization dose or ‘NA’. When many fertilization doses are reported for a given fertilization nutrient, all fertilization doses are added for the given fertilization nutrient.
7	Fertilization	176	Fertilization_NPK_Dose_Unit	Class	Unit of fertilization dose or ‘NA’.
7	Fertilization	177	Fertilization_NPK_Dose_Unit_Type	Class	Type of unit of fertilization dose or ‘NA’.
7	Fertilization	178	Fertilization_NPK_Dose_Product_Name	Class	Product name(s) of fertilization dose or ‘NA’.
7	Fertilization	179	IDCrop_Crop	Index	Corresponding index from the 'Crop' table. Secondary key of the 'Fertilization' table.
8	Weed_Insect_Fungi	180	IDPDW	Index	Index of each weed and/or insects and/or fungi management from each crop. Primary key of the 'Weed_Insect_Fungi' table.
8	Weed_Insect_Fungi	181	Weed_Insect_Fungi_Presence_Weed	Binary	There is ('1') or there is not ('0') weed management or ‘NA’. This attribute is mutually exclusive with the 'Weed_Insect_Fungi_Presence_Insect' and 'Weed_Insect_Fungi_Presence_Fungi' attributes.
8	Weed_Insect_Fungi	182	Weed_Insect_Fungi_Presence_Insect	Binary	There is ('1') or there is not ('0') insect management or ‘NA’. This attribute is mutually exclusive with the 'Weed_Insect_Fungi_Presence_Weed' and 'Weed_Insect_Fungi_Presence_Fungi' attributes.
8	Weed_Insect_Fungi	183	Weed_Insect_Fungi_Presence_Fungi	Binary	There is ('1') or there is not ('0') fungi management or ‘NA’. This attribute is mutually exclusive with the 'Weed_Insect_Fungi_Presence_Weed' and 'Weed_Insect_Fungi_Presence_Insect' attributes.
8	Weed_Insect_Fungi	184	Weed_Insect_Fungi_Presence_Treatment_Mechanical	Binary	If there is weed or insect or fungi management, then there is ('1') or there is not ('0') mechanical treatment. Elsewhere, ‘NA’ is reported.
8	Weed_Insect_Fungi	185	Weed_Insect_Fungi_Presence_Treatment_Mechanical_Date	Date	If there is mechanical treatment, then the date is reported or ‘NA’. If there is no mechanical treatment, then 'NULL' is reported.
8	Weed_Insect_Fungi	186	Weed_Insect_Fungi_Presence_Treatment_Chemical	Binary	If there is weed or insect or fungi management, then there is ('1') or there is not ('0') chemical treatment. Elsewhere, ‘NA’ is reported.
8	Weed_Insect_Fungi	187	Weed_Insect_Fungi_Presence_Treatment_Chemical_Date	Date	If there is chemical treatment, then the date is reported or ‘NA’. If there is no chemical treatment, then 'NULL' is reported.
8	Weed_Insect_Fungi	188	Weed_Insect_Fungi_Presence_Treatment_Chemical_Dose	Numerical	If there is chemical treatment, then the chemical dose is reported or ‘NA’. If there is no chemical treatment, then 'NULL' is reported.
8	Weed_Insect_Fungi	189	Weed_Insect_Fungi_Presence_Treatment_Chemical_Dose_Unit	Class	If there is chemical treatment, then the unit of chemical dose is reported or ‘NA’. If there is no chemical treatment, then 'NULL' is reported.
8	Weed_Insect_Fungi	190	Weed_Insect_Fungi_Presence_Treatment_Chemical_Dose_Unit_AI	Binary	If there is chemical dose, then the unit of chemical dose is reported ('1') or is not reported ('0') in active ingredients or ‘NA’. If there is no chemical treatment, then 'NULL' is reported.
8	Weed_Insect_Fungi	191	Weed_Insect_Fungi_Presence_Treatment_Chemical_Dose_Product_Name	Class	If there is chemical treatment, then the product name(s) of chemical dose is(are) reported or ‘NA’. If there is no chemical treatment, then 'NULL' is reported.
8	Weed_Insect_Fungi	192	IDCrop_Crop	Index	Corresponding index from the 'Crop' table. Secondary key of the 'Weed_Insect_Fungi' table.
9	Irrigation	193	IDIrrigation	Index	Index of each irrigation management from each crop. Primary key of the 'Irrigation' table.
9	Irrigation	194	Irrigation_Presence_Irrigation	Binary	There is ('1') or there is not ('0') irrigation management or ‘NA’.
9	Irrigation	195	Irrigation_Presence_Irrigation_Dose	Numerical	If there is irrigation management, then the irrigation dose is reported. If there is no irrigation management, then '0' is reported. Elsewhere, ‘NA’ is reported.
9	Irrigation	196	Irrigation_Presence_Irrigation_Dose_Unit	Class	If there is irrigation dose, then the unit of irrigation dose is reported. If there is no irrigation dose, then 'mm' is reported. Elsewhere, ‘NA’ is reported.
9	Irrigation	197	Irrigation_Presence_Irrigation_Method	Class	If there is irrigation management, then the irrigation method is reported. If there is no irrigation management, then 'NULL' is reported. Elsewhere, ‘NA’ is reported.
9	Irrigation	198	IDCrop_Crop	Index	Corresponding index from the 'Crop' table. Secondary key of the 'Irrigation' table.
The number of attributes, their names, types and definitions are presented for each table.					
